# Evaluation of Adsorption Ability of Lewatit^®^ VP OC 1065 and Diaion™ CR20 Ion Exchangers for Heavy Metals with Particular Consideration of Palladium(II) and Copper(II)

**DOI:** 10.3390/molecules29184386

**Published:** 2024-09-15

**Authors:** Anna Wołowicz, Zbigniew Hubicki

**Affiliations:** Department of Inorganic Chemistry, Faculty of Chemistry, Institute of Chemical Sciences, Maria Curie Sklodowska University, Maria Curie-Sklodowska Square 2, 20-031 Lublin, Poland; zbigniew.hubicki@mail.umcs.pl

**Keywords:** ion exchangers, adsorption, removal, palladium, copper, heavy metals, Lewatit^®^ VP OC 1065, Diaion™ CR20

## Abstract

The adsorption capacities of ion exchangers with the primary amine (Lewatit^®^ VP OC 1065) and polyamine (Diaion™ CR20) functional groups relative to Pd(II) and Cu(II) ions were tested in a batch system, taking into account the influence of the acid concentration (HCl: 0.1–6 mol/L; HCl-HNO_3_: 0.9–0.1 mol/L HCl—0.1–0.9 mol/L HNO_3_), phase contact time (1–240 min), initial concentration (10–1000 mg/L), agitation speed (120–180 rpm), bead size (0.385–1.2 mm), and temperature (293–333 K), as well as in a column system where the variable operating parameters were HCl and HNO_3_ concentrations. There were used the pseudo-first order, pseudo-second order, and intraparticle diffusion models to describe the kinetic studies and the Langmuir and Freundlich isotherm models to describe the equilibrium data to obtain better knowledge about the adsorption mechanism. The physicochemical properties of the ion exchangers were characterized by the nitrogen adsorption/desorption analyses, CHNS analysis, Fourier transform infrared spectroscopy, the sieve analysis, and points of zero charge measurements. As it was found, Lewatit^®^ VP OC 1065 exhibited a better ability to remove Pd(II) than Diaion™ CR20, and the adsorption ability series for heavy metals was as follows: Pd(II) >> Zn(II) ≈ Ni(II) >> Cu(II). The optimal experimental conditions for Pd(II) sorption were 0.1 mol/L HCl, agitation speed 180 rpm, temperature 293 K, and bead size fraction 0.43 mm ≤ f3 < 0.6 mm for Diaion™ CR20 and 0.315–1.25 mm for Lewatit^®^ VP OC 1065. The maximum adsorption capacities were 289.68 mg/g for Lewatit^®^ VP OC 1065 and 208.20 mg/g for Diaion™ CR20. The greatest adsorption ability of Lewatit^®^ VP OC 1065 for Pd(II) was also demonstrated in the column studies. The working ion exchange in the 0.1 mol/L HCl system was 0.1050 g/mL, much higher compared to Diaion™ CR20 (0.0545 g/mL). The best desorption yields of %*D*_1_ = 23.77% for Diaion™ CR20 and 33.57% for Lewatit^®^ VP OC 1065 were obtained using the 2 mol/L NH_3_·H_2_O solution.

## 1. Introduction

**Figure 1:**
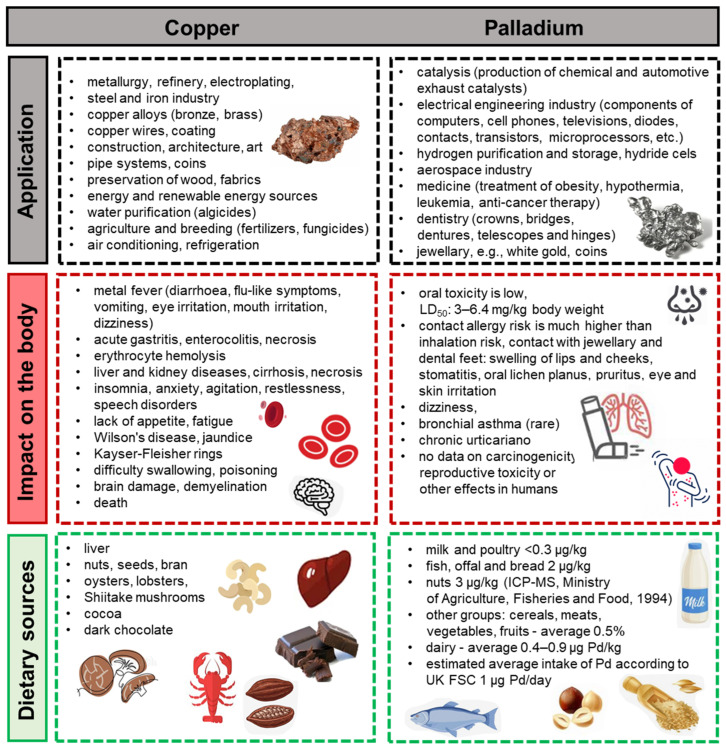

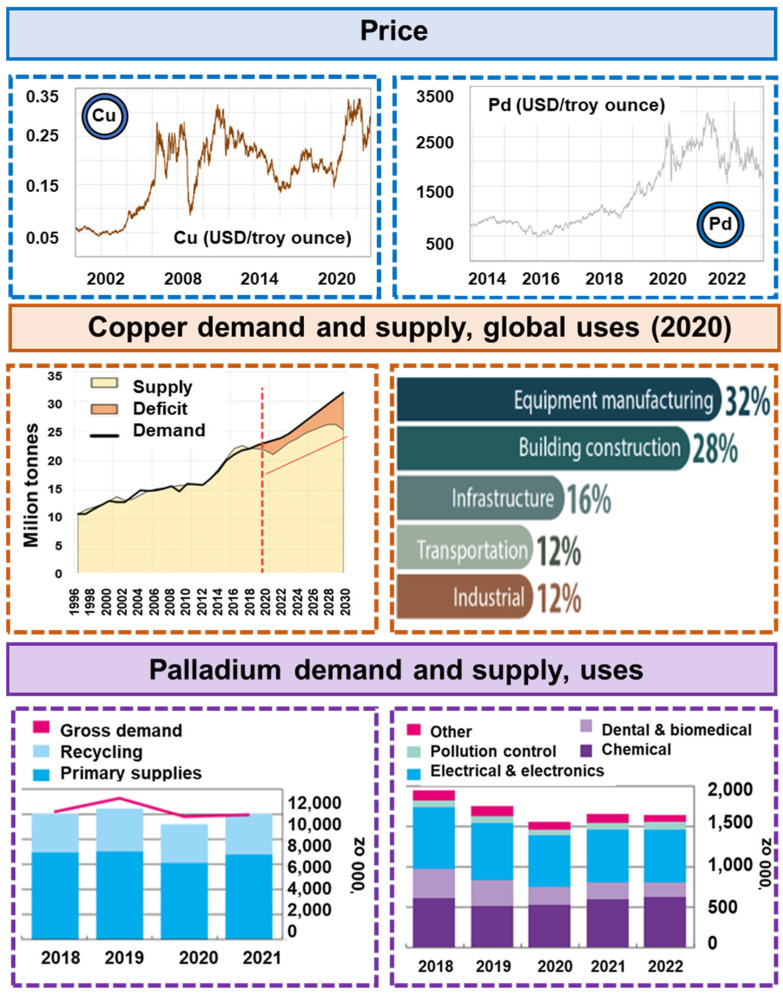
Palladium and copper application, impact on the body, dietary sources and prices, supply, demand, and uses.

Metals are widely used in various industries, and the rapid development of advanced technologies results in the fact that they play an increasingly important role in the global economy [1]. Palladium and copper are metals among the most important economical raw materials that are levers for the further development of civilization and are included in the category of crucial, strategic, and critical raw materials [2,3]. Due to their unique properties, copper and palladium are essential for many economic sectors, including transportation, electronic information, chemical industry production, infrastructure, and consumption products (Figure 1) [1,4]. In recent years, consumption of these metals in different branches of industry has grown rapidly, causing their demand to be larger than the supply, raising concern regarding the future availability of copper and palladium [4,5]. In addition to the resource availability concerns, there is increasing concern related to the energy requirement to produce these metals due to the fact that the mining industry is one of the most energy-intensive industrial sectors and one of the largest emitters of global CO_2_, which impacts the environment negatively [1]. Therefore, much attention is paid to copper and palladium recovery from their secondary sources, e.g., e-waste [6], waste catalysts [7], and anodic slimes [8], but these metals’ recovery is still at a low level and does not fill the demand gap. On the other hand, the increasing concentration of heavy metals in the environment due to their widely spread uses results in environmental pollution. The persistence of heavy metals in the environment and their ability to migrate and accumulate in the food chain, as well as their toxic properties, make them a particular threat to human health and the environment. The toxicity of heavy metals depends on such factors as dose, chemical species, route of exposure, gender, age, and genetics, as well as the nutritional status of exposed individuals [9].

Thus, it is necessary to search ways to remove them from wastewaters, often in combination with recovery of valuable metals.

Wastes, including palladium and copper metals, can be recovered via pyro- and hydrometallurgy routes. They are dissolved by acidic leaching without or with the presence of oxidants [10]. The widely used reagents for metal leaching are aqua regia, hydrochloric acid, sulfuric acid, a mixture of acids with oxygen gas or hydrogen peroxide as the oxidant, etc. [10,11,12]. Precipitation by increasing pH to remove heavy metals is a common practice as a purification process, but it generates sludges with greater potential risk for the environment, uses significant amounts of reagents for pH control, and results in a loss of valuable metals during iron removal by precipitation. Therefore, selective recovery of valuable, strategic metals prior to precipitation is desirable [11]. Adsorption processes are of considerable interest in palladium or/and copper hydrometallurgy. The use of polymeric resins with many beneficial properties and the comparison of capital and operating costs indicate that ion exchange resins can compete successfully with other recovery methods and can be considered as a feasible alternative during the design of palladium and copper hydrometallurgical recovery plants [10,13,14]. The ideal adsorbents should be characterized by a large accessible surface area, selectivity towards target heavy metals, strong interactions between active sites and removed metals, as well as easy regeneration [15]. Ion exchangers satisfy such recommendations, and as the literature data indicate, they are able to effectively remove palladium or copper with different efficiency levels [10,14]. Due to the fact that, after the leaching process of waste materials, precious metals are usually present in the form of anionic complexes, their removal using anion exchange resins with different basicity of their functional groups seems to be very promising, similarly to the application of chelating ion exchange resins, which are capable of selective removal of a given impurity [16]. In addition, the chelating ion exchangers are characterized by better selectivity, sensitivity, and adsorption capacity compared to the conventional ion exchange resins, especially for trace concentrations of heavy metal ions [16,17,18]. The chelating polymers containing N, O, and S donor atoms in their functional groups result in improved heavy metal removal and the required selectivity [18]. The chelating ion exchangers with iminodiacetic acid chelating groups are the most studied for heavy metal ion removal and separation, but the efficiency and versatility of other chelating exchangers have not been extensively discussed [18,19]. According to the manufacturer’s data sheet, Diaion™ CR20 has a highly porous matrix and is greatly recommended for the removal and recovery of divalent metal ions from wastewater. Due to the above-mentioned fact, in our studies, the chelating ion exchanger Diaion™ CR20 with the polyamine functional groups and the anionic Lewatit^®^ VP OC 1065 were selected. There is still a gap in the literature regarding the use of ion exchangers with different basicity of the functional groups for the removal of metal ions. Although such studies are carried out using the static method, they are very often incomplete, and studies using the dynamic method are usually neglected, although they are the basis for further studies on the use of ion exchangers for industrial applications. In order to design technologies for metal ion removal from aqueous solutions (wastewaters, solutions obtained after waste leaching), it is necessary to take into account not only the physicochemical properties of ion exchangers (the ion exchanger’s type of matrix, structure, functional groups), but also the experimental condition, such as the solution pH, adsorbent dose, ion exchanger bead size, initial concentration, agitation speed, phase contact time, temperature, adsorbate properties (type, chemical form, concentration), etc. [20,21]. Therefore, the purpose of this study was to evaluate the effectiveness of Pd(II) and Cu(II) ion removal from the aqueous solutions using Lewatit^®^ VP OC 1065 and Diaion™ CR20 ion exchange resins. Discussion on the ion exchanger properties not studied in the literature so far regarding to the results of the reaction rate and factors influencing the palladium and copper adsorption processes (acids concentration, HCl: 0.1–6 mol/L; HCl-HNO_3_: 0.9–0.1 mol/L HCl—0.1–0.9 mol/L HNO_3_; phase contact time, 1–240 min; initial concentration, 10–1000 mg/L; agitation speed, 120–180 rpm; bead size, 0.300–1.25 mm; temperature, 293–333 K) is a novel aspect of this paper. In addition, this approach will allow for a better understanding the reaction mechanisms as well as allow us to determine the potential of the tested Lewatit^®^ VP OC 1065 and Diaion™ CR20 ion exchangers for the selected metal ions.

## 2. Results and Discussion

### 2.1. Ion Exchange Resins Characterization

Lewatit^®^ VP OC 1065 and Diaion™ CR20 ion exchange resins were used for palladium(II) and copper(II) adsorption from the acidic solutions. The general description of these ion exchangers is presented in Section 3.1. Both ion exchange resins possess the polystyrene–divinylbenzene matrix and belong to the anion exchange and chelating resin types. Lewatit^®^ VP OC 1065 possesses the primary amine (benzylamine) functional groups, whereas Diaion™ CR20 possesses the polyamine ones. The specifications of these materials are presented in Table 1.

**Figure 2:**
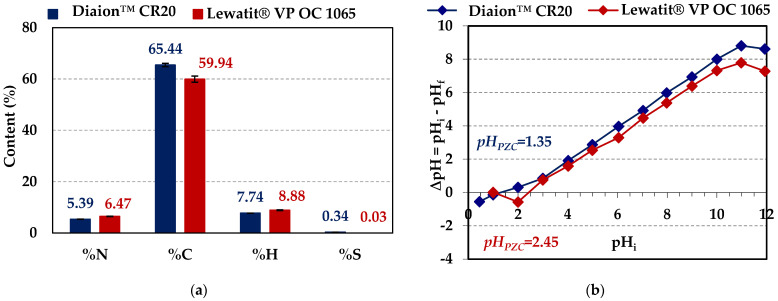
(**a**) Percentage content of elements and (**b**) comparison of *pH_PZC_* values in/for Lewatit^®^ VP OC 1065 and Diaion™ CR20 ion exchange resins.

The CHNS analysis for the pure (before adsorption) Lewatit^®^ VP OC 1065 and Diaion™ CR20 ion exchange resins was performed. The mean results of three measurements are presented in Figure 2a.

Slightly larger values of %N and %H content were observed for Lewatit^®^ VP OC 1065, whereas Diaion™ CR20 possessed higher contents of %C and %S.

Knowledge of the point of zero charge (*pH_PZC_*) value of adsorption materials is very important because it allows one to hypothesize about the ion exchangers’ functional group ionization and their interactions with heavy metal species in the aqueous solutions, and in the further perspective to determine their effect on adsorption efficiency. The *pH_PZC_* value of the adsorbent provides information about the possible attraction/repulsion between the adsorbent and the adsorbate [27,28,29]. Therefore, the point of zero charge for Lewatit^®^ VP OC 1065 was obtained previously (2.45) [26], and it was obtained for Diaion™ CR20 (1.35) in this paper using the pH-drift method (Figure 2b) [30]. The comparison of these values is presented in Figure 2b. The *pH_PZC_* value refers to the point where the plotted curve crosses the initial pH_i_. It is well known that when pH < *pH_PZC_*, cation adsorption is favorable, whereas when pH > *pH_PZC_*, anion sorption takes place [27].

The structural properties such as the specific surface area (*S_BET_*), the total pore volumes (*V_tot_*), and the average pore diameter (*D*) of the ion exchangers under discussion were determined and are presented in Table 2.

**Figure 3:**
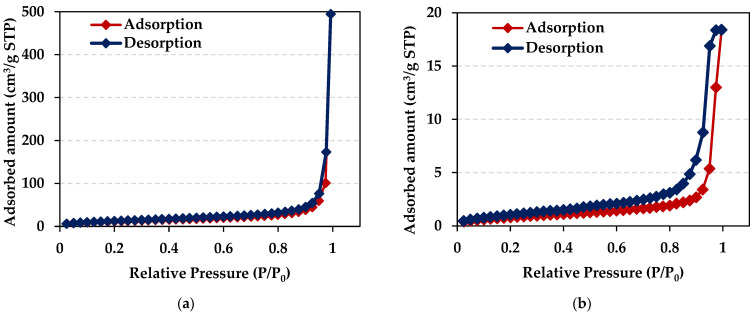
Low-temperature adsorption/desorption nitrogen isotherm of (**a**) Diaion™ CR20 and (**b**) Lewatit^®^ VP OC 1065 ion exchangers.

Taking into account the textural parameters, both ion exchangers possess a porous structure, but larger pore volumes and an average pore diameter were observed in the case of Diaion™ CR20 (micropores—less than 2 nm, mesopores—2 to 50 nm, and macropores—larger than 50 nm). Figure 3 shows the low-temperature nitrogen adsorption/desorption isotherms for Diaion™ CR20 and Lewatit^®^ VP OC 1065 ion exchange resins. The isotherms of Diaion™ CR20 and Lewatit^®^ VP OC 1065 can be categorized as Type II (the IUPAC classification) [31], indicating a mesoporous material [32]. The H3 hysteresis loop was observed in relative pressure being between 0.9 and 1.0 for Diaion™ CR20 and between 0.4 and 0.9 in the case of Lewatit^®^ VP OC 1065. 

**Figure 4:**
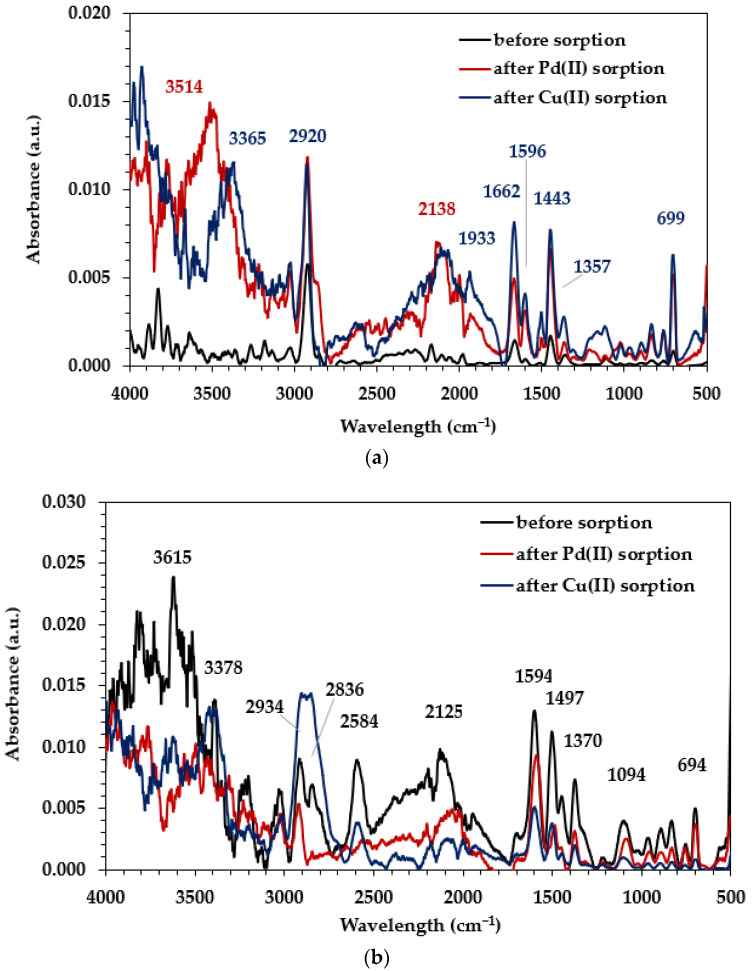
ATR/FT-IR spectra of (**a**) Diaion™ CR20 and (**b**) Lewatit^®^ VP OC 1065 before and after loading with Pd(II) and Cu(II) ions.

The ATR-FTIR spectra of the ion exchangers Diaion™ CR20 and Lewatit^®^ VP OC 1065 before and after Pd(II) and Cu(II) adsorption were recorded (Agilent Cary 630 FT-IR spectrometer) in the wavelength range of 500–4000 cm^−1^ to identify the ion exchange resins’ functional groups (raw ion exchangers) and to obtain knowledge about possible interactions between the functional groups and M(II) ions during the adsorption process (possible mechanism of the M(II) binding; loaded ion exchangers) using the Micro Lab FTIR software (Figure 4). Based on the raw spectra of ion exchangers, the band peaks at about 3600–3200 cm^−1^ related to the stretching vibrations of the O-H groups and the N-H groups in the case of both ion exchangers were observed [33,34]. In the range of 3090–3000 cm^−1^, bands corresponding to the stretching vibrations of the C-H groups were observed, whereas in the range 3000–2850 cm^−1^, the bands were assigned to the symmetric and asymmetric stretching vibrations originating from the aliphatic CH_2_ groups [35,36]. Both ion exchangers possessed the polystyrene–divinylbenzene matrix; therefore, the bands corresponded to the presence of the C=C group in the benzene ring—the symmetric stretching vibrations of the aromatic rings in the wavenumber range of 1610–1500 cm^−1^ were also confirmed [35,36]. The presence of low-intensity bands correlated to the deformation vibrations of the CH_2_ groups and stretching C-C in the range of 1215–1150 cm^−1^. Additionally, peaks corresponding to the amine functional groups of ion exchangers were found. The bands appearing in the ranges of 1650–1500 cm^−1^ and 910–660 cm^−1^ corresponded to the deformation vibrations of N-H groups, whereas those assigned to the stretching vibration of the C-N groups in the aliphatic or aromatic amine at 1250–1020 cm^−1^ and 1360–1250 cm^−1^ were found [36,37]. The comparison of the ATR-FTIR spectra of raw ion exchangers with the spectra of the Diaion™ CR20 and Lewatit^®^ VP OC 1065 after Pd(II) and Cu(II) ion adsorption (loaded ion exchangers) showed significant differences in the signal positions—some peaks moved away or their intensity changed after the M(II) adsorption. The peaks’ intensity usually increases after the M(II) ion adsorption on Diaion™ CR20, whereas it usually decreases after the M(II) ion adsorption on Lewatit^®^ VP OC 1065. The greatest changes were found in the range corresponding to the vibrations of the amino groups present in the functional group of the ion exchanger under discussion. These changes can indicate the ongoing M(II) adsorption process with participation of their functional groups [37].

### 2.2. Batch Adsorption Method—Experimental Conditions Effect on Pd(II) and Cu(II) Adsorption

The effects of different experimental conditions on selected heavy metal ions adsorption efficiency on Diaion™ CR20 and Lewatit^®^ VP OC 1065 ion exchangers were investigated.

The effects of acid concentration such as HCl (0.1 mol/L HCl (S1), 1 mol/L HCl (S2), 3 mol/L HCl (S3), 6 mol/L HCl (S4)), HCl–HNO_3_ (0.1 mol/L HCl–0.9 mol/L HNO_3_ (S5), 0.2 mol/L HCl–0.8 mol/L HNO_3_ (S6), 0.5 mol/L HCl–0.5 mol/L HNO_3_ (S7), 0.8 mol/L HCl–0.2 mol/L HNO_3_ (S8), 0.9 mol/L HCl–0.1 mol/L HNO_3_ (S9)), and the phase contact time (0–240 min) on the M(II) ion removal efficiency were compared. The amount of M(II) adsorption (*q_t_*, mg/g), the adsorption capacities (*q_e_*, mg/g), and the percentage removal (*R*, %) were calculated according to the following formulae:

- The amount of M(II) ions sorbed after time *t* (*q_t_*) (mg/g) per weight unit of sorbent under the non-equilibrium conditions:

(1) $$q_{t}=\frac{{(C}_{0}-C_{t})*V}{m}$$

- The adsorption capacity (*q*_e_) (mg/g):

(2) $$q_{e}=\frac{{(C}_{0}-C_{e})*V}{m}$$

**Figure 5:**
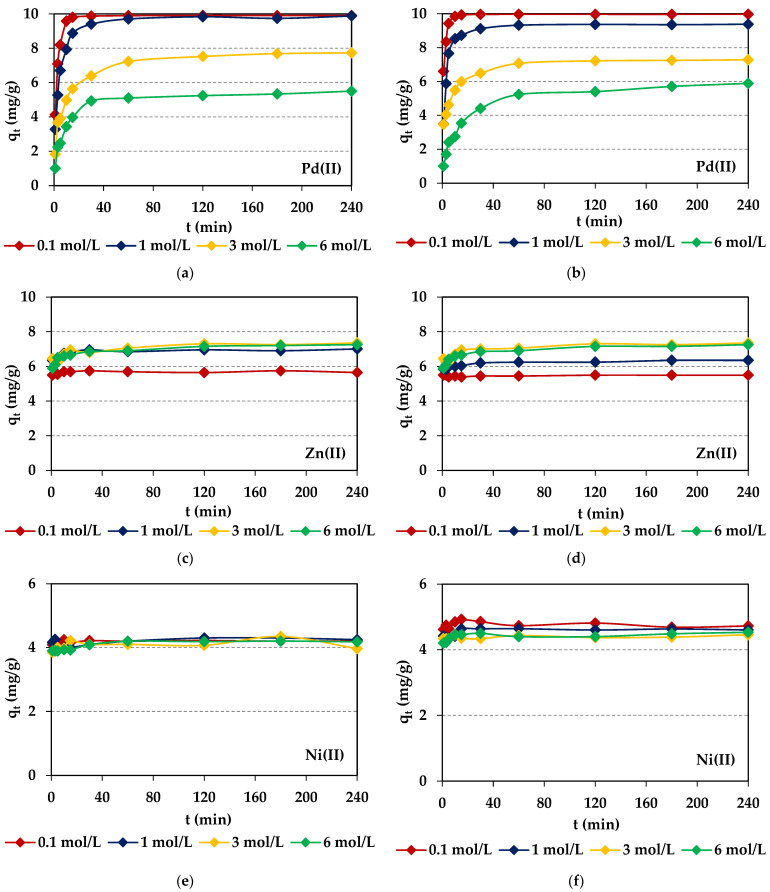

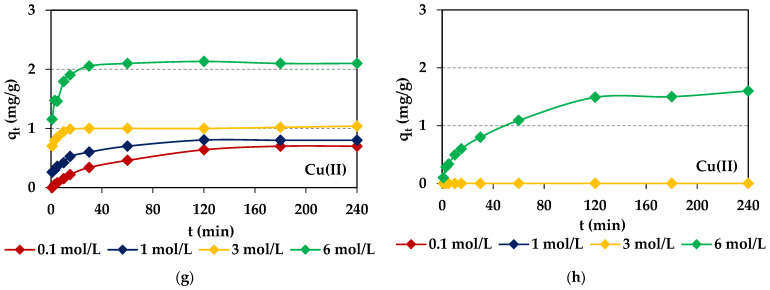
Comparison of M(II) sorption efficiency expressed in *q_t_* values for Diaion™ CR20 (**a**,**c**,**e**,**g**) and Lewatit^®^ VP OC 1065 (**b**,**d**,**f**,**h**).

- The percentage removal (*R*) (%):

(3) $$R=\frac{C_{0}-C_{t}}{C_{0}}*100\%$$

where: *C*_0_, *C_e_*—the initial and equilibrium concentrations of M(II) in the water phase (mg/L), *C_t_*—the concentration of M(II) in the water phase after time *t* (mg/L), *V*—the volume of the solution, and *m*—the dry ion exchanger mass. The course of the adsorption process in 240 min for different HCl concentrations and 100 mg M(II)/L (M(II)–Pd(II), Cu(II), Ni(II), and Zn(II)) is shown in Figure 5, whereas the results for the HCl-HNO_3_ systems are shown in Figure S1. The screening test shows that the metal ions adsorption efficiencies depend on the ion exchanger properties as well as the acid concentration, phase contact time, and type of metal. For both ion exchangers, the selectivity series is as follows: Pd(II) > Zn(II) > Ni(II) > Cu(II) in both the chloride and chloride-nitrate(V) systems. The largest adsorption capacities and percentage removal were obtained for the S1 system and Pd(II), which were equal to *q_e_* = 9.97 mg/g (*R* = 99.75%) for Lewatit^®^ VP OC 1065 and *q_e_* = 9.91 mg/g (*R* = 99.10%) for Diaion™ CR20. The adsorption capacities and percentage removal decrease with the HCl concentration increased from 0.1 to 6 mol/L (Diaion™ CR20: *q_e_* = 9.91 mg/g (*R* = 99.10%), S1 > *q_e_* = 9.88 mg/g (*R* = 98.80%), S2 > *q_e_* = 7.73 mg/g (*R* = 77.33%), S3 > *q_e_* = 5.88 mg/g (*R* = 58.76%), S4 and Lewatit^®^ VP OC 1065: *q_e_* = 9.97 mg/g (*R* = 99.75%), S1 > *q_e_* = 9.38 mg/g (*R* = 93.83%), S2 > *q_e_* = 7.29 mg/g (*R* = 72.85%), S3 > *q_e_* = 5.89 mg/g (*R* = 58.91%), S4). Diaion™ CR20 showed slightly higher values of *q_e_* and *R* for the S2 and S3 systems compared to Lewatit^®^ VP OC 1065, which indicates that here, the HCl effect was less visible. Both ion exchangers showed potential applications for Pd(II) removal from the dilute acidic solutions. Moreover, the time required to reach an equilibrium increased with the increasing HCl concentration. The percentage removal of Pd(II) was also the highest compared to that of other metal ions in the HCl-HNO_3_ systems (*t* = 240 min, *R* = 96.63–98.44% for Diaion™ CR20 and *R* = 92.81–98.19% for Lewatit^®^ VP OC 1065), and the adsorption capacities were in the range from 9.05 to 9.84 mg/g for both resins. Usually, higher M(II) adsorption efficiency was observed in the HCl than HCl-HNO_3_ solutions. The highest Zn(II) adsorption was observed for the 3 mol/L HCl solution, whereas that of Cu(II) was small, but it was the highest in the 6 mol/L HCl solution. In the HCl-HNO_3_ systems, copper was not practically adsorbed. In the case of Ni(II), there was a comparable adsorption efficiency in all systems in the whole adsorption time range, 1–240 min. Due to the fact that the Pd(II) adsorption efficiency was the largest compared to the other metal ions, the additional studies were carried out in this case. 

The studies on adsorption for removal of heavy metal ions from aqueous solutions indicate that the pH of the solution plays a particularly important role in both the heavy metal ion removal and separation processes [38,39,40]. The effect of pH on the M(II) adsorption process can be explained by the surface charge of the sorbent. The pH of the solution and the functional groups present on the surface of the adsorbent determine the nature and concentration of this charge [41]. The pH of the solution influences the ionization of the functional groups/active sites of the ion exchange resin and also the speciation of heavy metals in aqueous solutions [27,42]. As reported in the literature on acidity, the total chloride ion concentration of the aqueous chloride medium, aging of the solution, and temperature are the factors affecting the ionic species of PGMs in the solutions [43]. The metals complexes/species’ presence in the solutions depending on the total concentration of chloride ions, which affect the M(II) adsorption efficiency, is depicted in Figure S2, and it was described previously, e.g., for Pd(II), Cu(II), Zn(II), and Ni(II) [43,44]. In addition, the effect of pH on the M(II) species was described in the literature for Cu(II) [45], Ni(II) [46], Zn [47], and Pd(II) [10,48]. As noted, at the appropriate pH, e.g., above 6.5 for Cu(II) and Zn(II) or above 8 for Ni(II), precipitation of heavy metal ions begins, resulting in a reduced removal efficiency. Various aqueous and hydroxochloride complexes of different stabilities and kinetic inertness can be formed in aqueous solutions under the influence of the above-mentioned factors [40,43,44]. In the case of 0.1–6 mol/L HCl solutions, M(II) exists in the form of divalent cations and anionic, cationic, and neutral species. The dominant species of Pd(II) in [Cl]^−^ > 0.1 mol/L solutions are the anionic PdCl_4_^2−^ complexes. The aquachloro and aquahydroxo complexes also appear in relation to aging and temperature [32]. In the acidic chloride media, PdCl_4_^2−^, as an anionic species, is capable of undergoing anion-exchange reactions, and depending on the basicity of the ion exchanger, the mechanism of sorption will be ion association or chelation [10,49]. The general reaction of the anion exchange process can be expressed by the following reaction:

*n*B^+^X^−^ + [MX*_m_*]*^n^*^−^ ↔ [B]*_n_*[MX*_m_*] + *n*X^−^ (4)

where B is the organic bases; M is the palladium; and X is the ligand (chloride ions). For Pd(II) *m* = 4 and *n* = 2 [10,31,41,49], the palladium binding by the electrostatic interactions and chelation/coordination mechanism is as follows:

RNH + HCl_(aq)_ → RNH_2_^+^ + Cl^−^_(sorb)_ (5)

2RNH_2_^+^Cl^−^_(sorb)_ + [PdCl_4_]^2−^_(aq)_ → (RNH_2_^+^)_2_[PdCl_4_]^2−^_(sorb)_ + 2Cl^−^_(aq)_ (6)

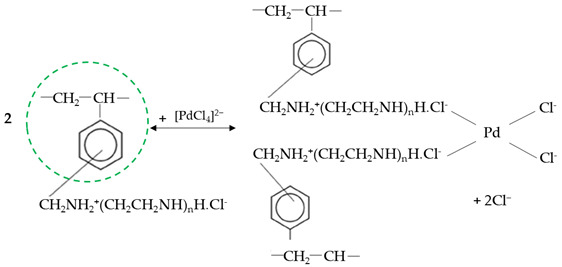
(7)

RNH_2_^+^Cl^−^_(sorb)_ + [PdCl_4_]^2−^_(aq)_ → RHN→[PdCl_3_]^−^_(sorb)_ + H^+^_(aq)_ + 2Cl^−^_(aq)_ (8)

The metal-chloro complexes tend to form ion pairs with anion exchangers, which are as follows: [MCl_6_]^2−^ > [MCl_4_]^2−^ > [MCl_6_]^3−^ > aqua species. The order is determined by the species charge/size ratio or charge density. The species with low charge density are more easily paired than those with a larger charge density. This is possibly a consequence of the size of the hydration shell surrounding the ions; densely charged species with larger hydration shells have fewer coulombic interactions with their counterions than those with smaller hydration shells. For example, the hydrated radius of Cu(II) is equal to 0.419 nm (ionic radius 0.073 nm), whereas that of Ni(II) is 0.404 nm (0.069 nm); therefore, Cu(II) adsorption is hindered [50]. Diaion™ CR20 and Lewatit^®^ VP OC 1065 possess chelating and/or weakly basic ion exchanger functionality. The weakly basic ion exchange resin groups protonate significantly in the acidic solutions; therefore, in strongly acidic solutions, this resin begins to work more effectively and the adsorption capacity is the highest. Electrostatic interactions between the anionic PdCl_4_^2−^ complexes and the positively charged functional groups can occur. The capability of Pd(II) adsorption on the weakly basic anion exchangers proceeds is due to chelating interactions rather than a conventional ion association process [10]. The decreasing adsorption ability of the Lewatit^®^ VP OC 1065 with the increasing HCl concentration is a consequence of the competition between the chloride ions and the PdCl_4_^2−^ metal-complex anion [39,43,50]. At a low concentration of HCl solution (0.5–2 mol/L), Zn(II) exists in the form of cationic ZnCl^+^; therefore, the Zn(II) adsorption is lower than in the more concentrated HCl solutions. Above the HCl concentration of 2 mol/L, the quantities of the anionic species ZnCl_3_^−^ and ZnCl_4_^2−^ predominate, whereas the cationic species fraction significantly decreases, which results in an increased adsorption ability of the anion exchanger resin by the anion exchange mechanism [51]:

2RCl + [ZnCl_4_]^2−^→R_2_[ZnCl_4_]^2−^ + 2Cl^−^ (9)

In the 6 mol/L HCl solution, competing effects, as for Pd(II), were observed; therefore, the adsorption of Zn(II) was the largest in the 3 mol/L HCl solutions [43,51]. In the system containing Ni(II), the main species in the chloride solution under discussion were cationic species such as Ni^2+^, NiCl^+^, and neutral NiCl_2_ ones, and their proportions were comparable. The NiCl^+^ concentration increased insignificantly with the increasing concentration of HCl. Then, the concentration of Ni^2+^ decreased, so the effect of the HCl concentration was negligible [52]. The diversify of Pd(II) and Cu(II) adsorption efficiency onto the chelating ion exchanger Diaion™ CR20, which can also show the weakly basic ion exchange functionality, can result from the Pearson’s hard–soft acid–base (HSAB) theory, in which Pd(II) soft metal ions prefer to coordinate with soft bases (S > N > O) and Cu(II), Zn(II), and Ni(II), as borderline metal ions, to hard bases (O > N > S) [43].

#### 2.2.1. Effect of Agitation Speed on Pd(II) Adsorption

**Figure 6:**
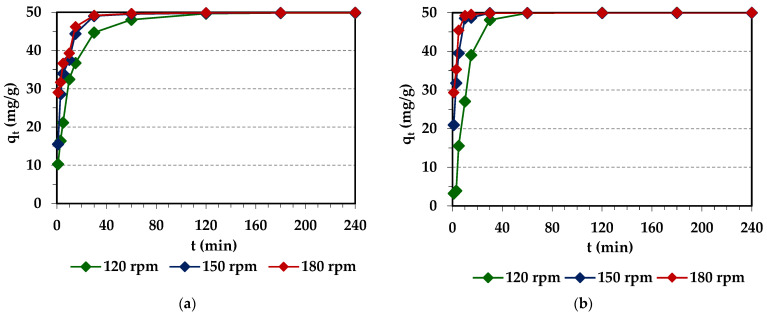
Effects of contact time and agitation speed on the Pd(II) adsorption on Diaion™ CR20 (**a**) and Lewatit^®^ VP OC 1065 (**b**).

Agitation speed is one of the important parameter effects on adsorption efficiency, and is mostly neglected by some researchers in adsorption studies [53]. The appropriate agitation speed increases the movement of metal ions in the mixture, which reduces the mass transfer resistance in the process. In batch adsorption systems, agitation speed affects the external boundary film and the distribution of the solute in the bulk solution [54]. Figure 6 depicts the effect of agitation speed on the amount of Pd(II) adsorbed on the ion exchangers under discussion at agitation speeds ranging from 120 to 180 rpm. It was observed that agitation speed influenced the adsorption efficiency of Pd(II). As the agitation speed increased from 120 to 180 rpm, a larger amount of Pd(II) was removed in a shorter time, e.g., for 10 min of phase contact time, the amount of Pd(II) adsorbed on Diaion™ CR20 increased from 32.47 mg/g (120 rpm) to 37.59 (150 rpm) to 39.29 mg/g (180 rpm), whereas on Lewatit^®^ VP OC 1065, it increased from 27.05 mg/g (120 rpm) to 48.56 (150 rpm) to 49.24 mg/g (180 rpm). The system reached equilibrium in a shorter time at a higher agitation speed, e.g., at 120 rpm, the time required to reach equilibrium was 180 min for Diaion™ CR20 and 120 min for Lewatit^®^ VP OC 1065, and shorter as the stirring speed increased to 180 rpm and was equal to 120 min for Diaion™ CR20 and 30 min for Lewatit^®^ VP OC 1065. Consequently, there was a rapid increase in metal ion removal for up to 60 min, and thereafter, despite different agitation speeds, the adsorption capacities (*q_e_*, mg/g) were at similar levels: 49.82 mg/g, 49.86 mg/g, and 49.89 mg/g for Diaion™ CR20 and 49.96 mg/g, 49.96 mg/g, and 49.97 mg/g for Lewatit^®^ VP OC 1065 at 120, 150, and 180 rpm agitation speeds, respectively.

A similar observation of agitation speed effects on the Pd(II) adsorption efficiency was previously reported during the Pd(II) adsorption on Dowex M4195 (Dow Chemical Company, Philadelphia, PA, USA) [43], Lewatit MonoPlus TP220 (Lanxess, Elmira, ON, Canada) [44], Lewatit MonoPlus SR7 [55] (Lanxess, Cologne, Germany), Dowex PSR2 and Dowex PSR3 (Dow Chemical Company, Philadelphia, PA, USA) [56], Purolite A400TL [57], and Purolite A830 (Purolite International Co, Philadelphia, PA, USA) [58] ion exchangers. A small effect of stirring speed (100–1400 rpm) on Pd(II) adsorption onto the magnetic chitosan nanoparticles (Fe_3_O_4_-CSN) was observed by Omidinasab et al. [59] (Table 3). In a solid–liquid adsorption process adsorbate transfer can be controlled by resistances to film diffusion (so called external diffusion), surface diffusion, and pore diffusion, or combined surface and pore diffusion. A higher agitation speed is supposed to decrease the boundary layer (resistance to film diffusion) and thus increase the speed at which the adsorption system reaches equilibrium [60]. At lower agitation speeds, the fluid film around the resin bead is thicker, whereas the boundary layer becomes thinner at higher agitation speeds, which influences the speed of transfer of metal ions through these layers. At lower agitation speeds, the film diffusion seems to be a rate-limiting step, and the adsorption kinetics of Pd(II) is influenced by small mass transfer of the adsorbate to the interior surface of the resin bead. In the case of a faster agitation speed, diffusion through the boundary layer increases, and the pore diffusion thus becomes a rate-limiting step [60]. The optimum agitation speed selected for future studies was 180 rpm.

#### 2.2.2. Effects of Initial Pd(II) Concentration

**Figure 7:**
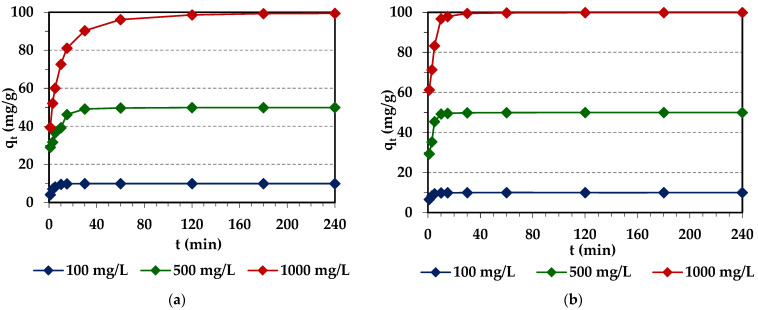
Effects of contact time and the initial Pd(II) concentration on Pd(II) adsorption on Diaion™ CR20 (**a**) and Lewatit^®^ VP OC 1065 (**b**).

Designing the adsorption system requires knowledge not only about the rate and efficiency of removing metal ions from the aqueous solutions, but also about the mechanism of adsorption. In order to determine the equilibrium capacity, studies were carried out at different phase contact times of 1–240 min and at various initial concentrations of the solutions of 0.1 mol/L HCl—x mg Pd(II)/L, where x—*C*_0_ = 100, 500, 1000 mg/L. The course of the adsorption process in 240 min for different initial concentrations of Pd(II) ions is shown in Figure 7. It can be seen that the initial Pd(II) concentration affects the shape of the kinetic curves and the Pd(II) adsorption efficiency. As the initial Pd(II) concentration increases, the amount of adsorbed Pd(II) increases and the equilibrium capacities are 9.91 mg/g (100 mg/L) > 49.89 mg/g (500 mg/L) > 99.41 mg/g (1000 mg/L) for Diaion™ CR20 and 9.97 mg/g (100 mg/L) > 49.97 mg/g (500 mg/L) > 99.87 mg/g (1000 mg/L) for Lewatit^®^ VP OC 1065. It can be seen that the kinetics of Pd(II) ion adsorption involves three steps. At the beginning (initial stage), the adsorption of Pd(II) is fast due to a large availability of free adsorption sites, and after that, it is slower up to the last stage, where equilibrium is achieved. The presence of adsorbed Pd(II) ions on the surface of the ion exchanger increases the repulsive forces, making the remaining sites more difficult to access [61,62]. The kinetic curves are less steep at higher Pd(II) initial concentrations, and the system equilibria are obtained after a longer period of time. The time required to reach an equilibrium increases from 30 min to 60 min (Lewatit^®^ VP OC 1065) and to 180 min (Diaion™ CR20) with the initial increase in Pd(II) concentration from 100 to 1000 mg/L. Lewatit^®^ VP OC 1065 is characterized by faster kinetics compared with the Diaion™ CR20 ion exchanger.

The percentage removal of Pd(II) is high for both ion exchangers, which are in the range from 99.1 to 99.79% for Diaion™ CR20 and from 99.75 to 99.94% for Lewatit^®^ VP OC 1065. The effects of the initial Pd(II) concentrations (*C*_0_ = 50–1000 mg/L) being equal to 50, 100, 200, and 300 mg/L for Purolite A830 [58] and Lewatit MonoPlus SR7 [55], as well as 100, 500, and 1000 mg/L for the Lewatit MonoPlus TP220 [44], Dowex PSR2, Dowex PSR3 [56], and Purolite A400TL [57] ion exchangers and Lewatit AF5 sorbent [60], respectively, on Pd(II) adsorption efficiency were previously examined. The results obtained in the chloride (0.1 mol/L HCl—x mg Pd(II)/L, S1) and chloride-nitrate(V) systems (0.9 mol/L HCl–0.1 mol/L HNO_3_—x mg Pd(II)/L, S9), where x means *C*_0_ = 50–1000 mg/L, indicate that: (1) The amount of adsorbed Pd(II) ions increases with the increasing phase contact time and with the increasing initial concentration of Pd(II) ions in all studied systems. (2) The values of the adsorption capacities increase with the increasing initial concentration of Pd(II) ions in the S1 and S9 solutions (except for Dowex PSR2, S9), e.g., from 5 to 30 mg/g (S1) and from 4.87 to 28.96 mg/g (S9) for Purolite A830 [58], from 10 to 99.95 mg/g (S1) for Lewatit MonoPlus TP220 [44], etc. (3) The time required to reach equilibrium (*t_eq_*) increases with the increasing initial concentration of Pd(II) ions and the increase in *t_eq_* evidenced especially in the S9 system. For example, for the anion exchange resin Purolite A830, the time increases from 15 to 30 min (S1) and from 60 min (50 mg/L and 100 mg/L) to 240 min (200 mg/L) to 360 min (300 mg/L) (S9) [58], while for the ion exchanger Lewatit MonoPlus TP220, it increases from 10 min (100 mg/L) to 120 min (1000 mg/L) (S1) [44], etc. Such results and the observed tendency are consistent with the results obtained in this paper.

#### 2.2.3. Effects of Ion Exchangers Bead Size

**Figure 8:**
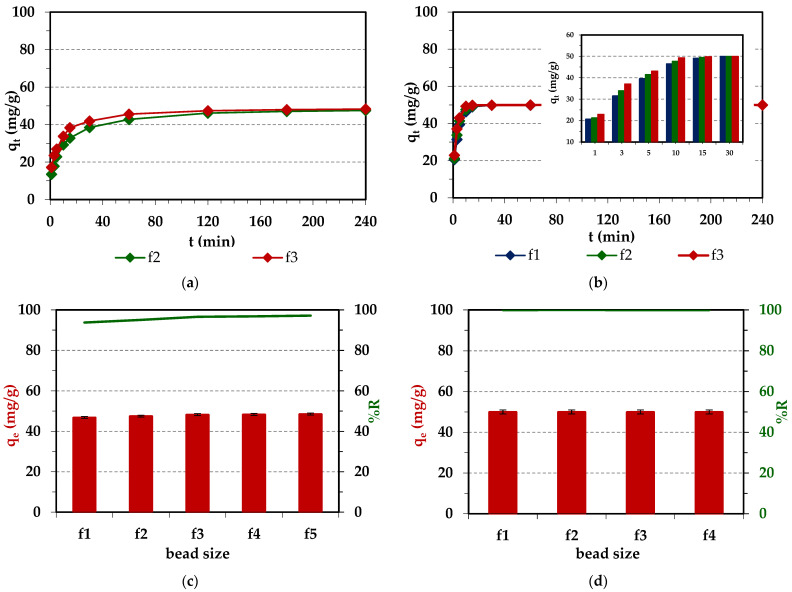
Effects of contact time and bead size of ion exchangers (f5 < 0.385 mm; 0.385 mm ≤ f4 < 0.43 mm; 0.43 mm ≤ f3 < 0.6 mm; 0.6 mm ≤ f2 < 0.75 mm; 0.75 mm ≤ f1 < 1.2 mm) on Pd(II) adsorption on Diaion™ CR20 (**a**,**c**) and Lewatit^®^ VP OC 1065 (**b**,**d**).

Due to the heterodispersive properties of ion exchangers, the effects of the bead size on the efficiency and removal rate of Pd(II) ions were also investigated (Figure 8). The commercial heterodispersive ion exchangers obtained by the suspension polymerization are characterized by the bead sizes in the range of 0.3–1.2 mm and the uniformity coefficient UC = 1.5–1.9, while the monodisperse ion exchangers have bead sizes in the range of 0.6–0.8 mm and UC = 1.1–1.2. More than 95% of the bead fractions of monodispersive ion exchangers differ slightly in diameter, or this difference is small (up to 0.05 mm) [63].

Particle size and size distribution have a direct effect on the adsorption material properties, which determine its potential application. The surface and textural properties of the adsorption material can influence the adsorption of heavy metal ions. Both ion exchangers Lewatit^®^ VP OC 1065 and Diaion™ CR20 have beads of different sizes (50 g), which were divided into fractions (f1–f5) by sieve analysis (Figure S3a,c). It was found that Lewatit^®^ VP OC 1065 has a slightly greater amount of larger-sized ion exchange beads compared to Diaion™ CR20 (Figure S3b). The effects of bead size on the amount of adsorbed Pd(II) were evident for Lewatit^®^ VP OC 1065 within the first 15 min of phase contact time (*q_t_* values increase with the decreasing bead size). A similar trend was also observed for Diaion™ CR20 (Figure 8a,b). Moreover, the results showed that with the bead size decrease, the adsorption capacities at 240 min of phase contact time for Diaion™ CR20 and Lewatit^®^ VP OC 1065 were at similar levels for all fractions (*q_e_* values for Diaion™ CR20 were from 46.89 to 48.59 mg/g, and the *q_e_* value for Lewatit^®^ VP OC 1065 was 49.97 mg/g). Such a trend could be related to the surface area for the adsorption of ion exchangers, which increases with the decreasing bead size, which usually results in enhanced adsorption and could be related to the metal ion diffusivity and porosity properties of ion exchangers [64,65]. As the bead diameter decreased, the specific surface area of the adsorbent (adsorption area) and the number of accessible adsorption sites increased, resulting in increases in both the rate and efficiency of adsorption. In the case of small beads, the ions diffusing into the interior of the ion exchange resin must follow a different path than in the case of larger beads, so the diffusion rate is not independent of the resin radius. The step that determines the overall process rate can be intraparticle diffusion (inversely proportional to the square of the radius of the ion exchange beads) or film diffusion (inversely proportional to the radius of the bead size) [36]. In addition, the diffusion rate depends on the ratio between the bead surface area and the migration path, as well as on the concentration gradient [45]. The obtained results are in good correlation with those presented in other papers concerning the evaluation of the effects of the bead size of ion exchangers on the efficiency of heavy metal ion adsorption. For example, the effects of bead size on the amount of adsorbed Pd(II) were observed only at the beginning of Pd(II) adsorption on Purolite A-830 (weakly basic anion exchanger) [58] or Purolite A-400TL (strongly basic anion exchanger) [57]. 

#### 2.2.4. Effects of Temperature on Pd(II) Adsorption

**Figure 9:**
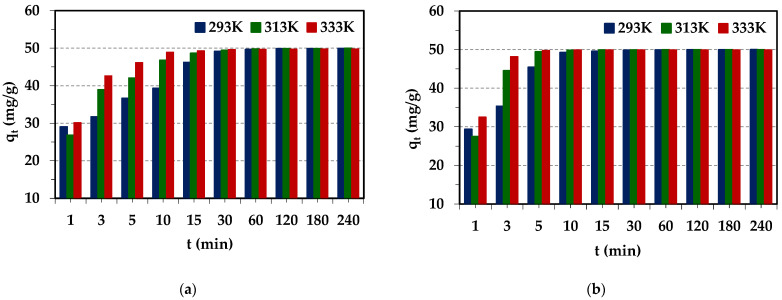
Effects of contact time and temperature on the Pd(II) adsorption on Diaion™ CR20 (**a**) and Lewatit^®^ VP OC 1065 (**b**).

The effects of temperature on the Pd(II) removal efficiency from the aqueous solution using the Diaion™ CR20 and Lewatit^®^ VP OC 1065 ion exchangers were studied. The results are depicted in Figure 9. 

The obtained adsorption capacities remained at similar levels with the increasing temperature for both ion exchangers. The amount of adsorbed Pd(II) ions increased with the temperature increasing from 293 to 313 K only in the initial 0–60 min stage of the adsorption process. The increase in temperature resulted in a decrease in the thickness of the boundary layer surrounding the ion exchanger beads and the increase in the ions mobility, which promoted the increased adsorption of Pd(II) ions. Additionally, the temperature increase can also alter the swelling of ion exchange resins and increase the diffusivity of ions in the ion exchangers pores [66]. As indicated by Sáncheaz et al. [67], the increase in temperature causes faster kinetics of the adsorption process of Pd(II) and Au(III) ions, and this effect is greater for Au(III) ions. As was observed in our case, the temperature increase shortened the time necessary to obtain system equilibrium, but did not affect the final adsorption capacities. Therefore, 293 K was selected as the optimal temperature. 

#### 2.2.5. Effects of Experimental Conditions on the Cu(II) Adsorption

**Figure 10:**
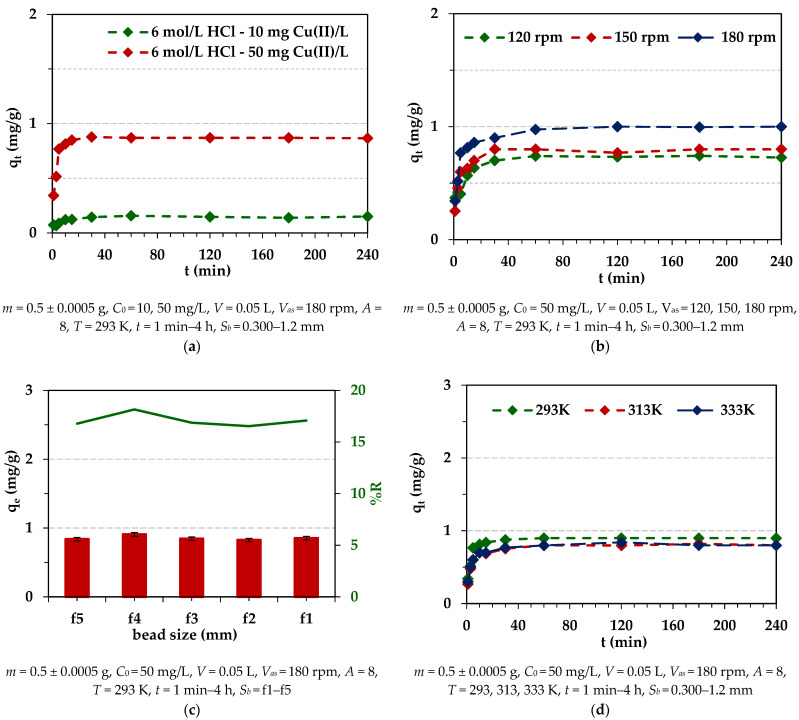
Effects of contact time and initial concentration (**a**), agitation speed (**b**), bead size of ion exchanger (f5 < 0.385 mm; 0.385 mm ≤ f4 < 0.43 mm; 0.43 mm ≤ f3 < 0.6 mm; 0.6 mm ≤ f2 < 0.75 mm; 0.75 mm ≤ f1 < 1.2 mm), (**c**) and temperature (**d**) on Cu(II) adsorption on Diaion™ CR20 from 6 mol/L HCl—10 (**a**) or 50 mg Cu(II)/L (**a**–**d**).

The adsorption of Cu(II) on ion exchangers was small (about 20%), but for Diaion™ CR20, it was slightly higher than in the case of Lewatit^®^ VP OC 1065. Therefore, for Diaion™ CR20, similar studies to those on Pd(II) were carried out to find whether the dependences were similar. Due to the largest adsorption efficiency of Cu(II) being achieved in the 6 mol/L HCl solution, this solution was selected for studies. The effects of different experimental conditions on Cu(II) adsorption efficiency are presented in Figure 10. 

As was found, the effects of the initial concentration and stirring speed on Cu(II) adsorption efficiency were the same as in the case of Pd(II) ions. Generally, the effects of bead size were small but slightly visible at the beginning of the adsorption process, similar to the observation for Pd(II). The effects of temperature were slightly visible. The amount of adsorbed Pd(II) ions increased with the increasing phase contact time at the beginning of the sorption process, and then at the equilibrium time, the adsorption capacities were at a similar level for all temperatures. In the literature, the increased adsorption of adsorbate with the increasing temperature is explained by the following assumptions: (1) with the increasing temperature, the diffusion rate of the adsorbate to the outer boundary layer and into the inner pores of the adsorbent increases, thus decreasing the viscosity of the solution; (2) with the increasing temperature, the mobility of the heavy metal ions increases; (3) as the temperature increases, the energy of the adsorbate can be increased, resulting in more interactions of the adsorbate with active sites on the surface; (4) at a higher temperature, the swelling effect of the internal porous structure of the adsorbent can take place, which may lead to the increasing ability of the adsorbate to penetrate into the pores of the adsorbent [68,69]. As pointed out in the literature, the significance of the temperature effect depends also on the type of adsorption mechanism. The adsorption mechanisms for inorganic pollutants involve strong adsorbate–adsorbent interactions. An increase in temperature could increase the energetics of this interaction, resulting in an increase in adsorption. The increase in adsorption with the increasing temperature indicated that adsorption of heavy metal ions by an adsorbent can involve not only physical, but also chemical adsorption [66]. Based on the literature data and chosen examples presented in Table S1 [70,71,72,73], the temperature effects on Cu(II) adsorption on different adsorbents are diverse. A low-temperature effect on Cu(II) adsorption was also observed by other researchers, which is consistent with our studies [73].

### 2.3. Kinetic Parameters for Pd(II) Adsorption 

Fast and effective adsorption plays a key role in industrial application. The kinetic studies determining the adsorption rate and kinetic parameters are important, since such parameters enable characterization of the movement of the sorbate within and onto the surface sites of the sorbent and make it possible to assess the applicability of a given adsorbent. For this purpose, the obtained adsorption data (Section 2.2) were modeled using the commonly known and applied kinetic models to describe the adsorption process. The pseudo-first-order (PFO) [74], the pseudo-second-order (PSO) [75], and the Weber–Morris (the intraparticle diffusion, IPD) [76] kinetic models were used and are presented in Table 4. The values of Marquardt’s percent standard deviation (MPSD), the determination coefficient (*R*^2^), and the adjusted R-squared ($R_{adj}^{2}$) were calculated to find the best kinetic model for adsorption data description.

Based on the PFO, PSO, and IPD models, the kinetic parameters for the Pd(II) were calculated and are presented in Table 5 for PSO and in Table S2 for PFO and IPD. The linear (LN) and non-linear (Non—LN) regression methods were applied. 

**Figure 11:**
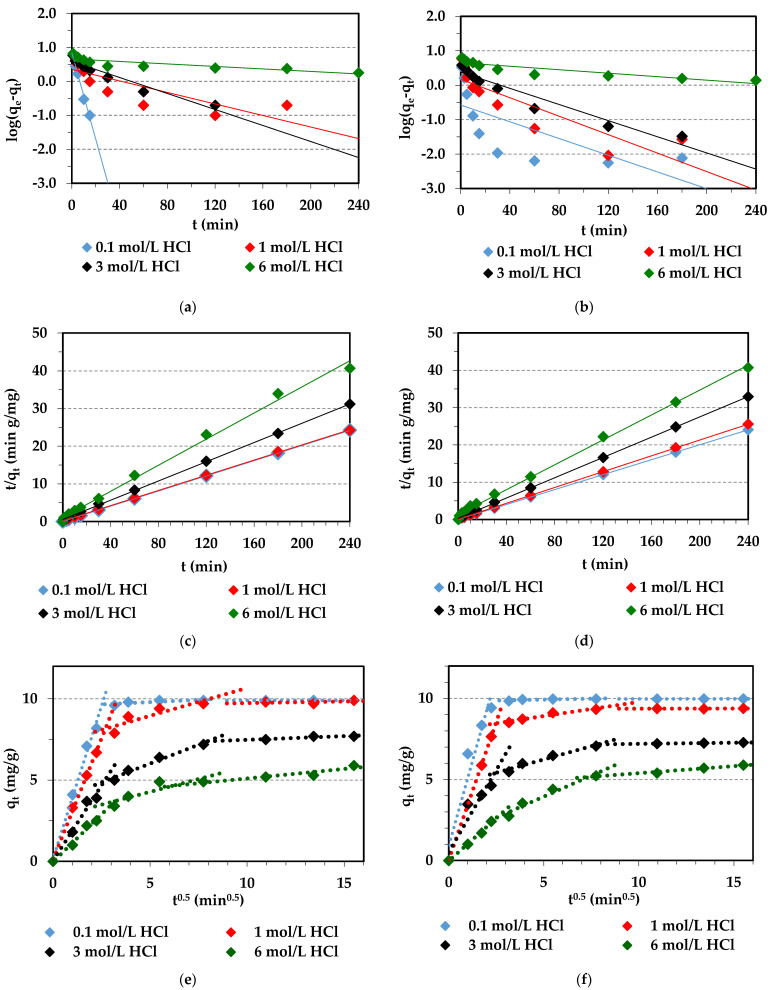

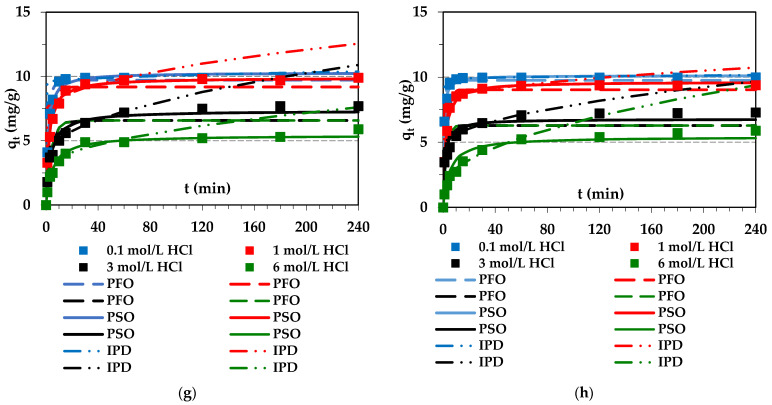
PFO (**a**,**b**), PSO (**c**,**d**), and IPD (**e**,**f**) plots and fitting of the experimental data of Pd(II) ion adsorption on Diaion™ CR20 (**g**) and Lewatit^®^ VP OC 1065 (**h**).

As follows from Table S2, the correlation coefficients (*R*^2^) for the PFO model were not high for Pd(II) or any HCl concentrations (*R*^2^ = 0.491–0.992 (L) and *R*^2^ = 0.560–0.971 (Non-LR)). Moreover, the determined *q_e cal_* values calculated from the PFO equation differed from the experimental ones (*q_e cal_*), indicating that the PFO kinetic model is not suitable for the Pd(II) adsorption process description (Figure 11a,b,g,h). 

On the other hand, the *R*^2^ values obtained for the PSO model were large in the HCl concentration range (*R*^2^ = 0.995–1.000 (L) and *R*^2^ = 0.873–0.991 (Non-LR)). Moreover, there was good agreement between the estimated values calculated from the PSO equation and the experimental ones. Additionally, the *t*/*q_t_* versus *t* plots of the PSO kinetic model (Figure 11c,d) were rectilinear over the entire range, indicating that the PSO model fit the data most satisfactorily. The initial adsorption rate, *h*, and the rate constants, *k*_2_, determined from the PSO equation were the largest in the 0.1 mol/L HCl solution and decreased significantly with the increasing HCl concentration up to 6 mol/L. This tendency can be attributed to the increasing concentration of Cl^−^ anions, which led to smaller adsorption rates and competition effects [40].

The rate-limiting step of Pd(II) adsorption can be distinguished, taking into consideration diffusion mechanisms (external diffusion, boundary layer diffusion, and intraparticle diffusion). The Weber–Morris plot (Figure 11e,f) shows the multi-stages of the adsorption process. The plot of *q_t_* vs. *t*^0^.^5^ indicates three linear portions. This implies the existence of three stages that contribute to the adsorption process: the Pd(II) diffusion from the solution to the outer surface of the ion exchanger; film diffusion, i.e., the Pd(II) movement from the boundary layer to the sorbent surface, adsorption on the particles external surfaces, then adsorption of Pd(II) by active adsorption sites in the sorbent interior pore spaces; and finally, intraparticle Pd(II) diffusion onto the sorbent pores [79,81]. It was found that the determination coefficients for the IPD model are smaller (*R*^2^ = 0.608–0.974) than for the PSO model and the plot does not pass through the origin, indicating that intraparticle diffusion is not a sole rate-limiting step. 

### 2.4. Isotherm Parameters for Pd(II) and Cu(II) Adsorption

Adsorption isotherms are useful for selecting the most appropriate adsorbent. They also play a crucial role in predicting the performance of adsorption systems. The equilibrium studies and obtained isotherms give insight into the maximum adsorption capacities. Moreover, determination of the equilibrium parameters and interpretation of the obtained isotherms provide valuable information about the nature of the metal ion–ion exchanger interactions and the adsorption mechanism taking place. In adsorption isotherm studies, frequently known isotherms such as the Langmuir [82] and Freundlich [83] ones have been employed to describe the equilibrium adsorption process and to fit the experimental data:

- The Langmuir model [82,84]:

(19) $$\mathrm{The}\;\mathrm{linear}\;\mathrm{form}\;\;\frac{C_{e}}{q_{e}}=\frac{1}{Q_{0}k_{L}}+\frac{C_{e}}{Q_{0}}$$

(20) $$\mathrm{The}\;\mathrm{non}-\mathrm{linear}\;\mathrm{form}\;q_{e}=\frac{k_{L}Q_{0}C_{e}}{1+C_{e}k_{L}}$$

**Figure 12:**
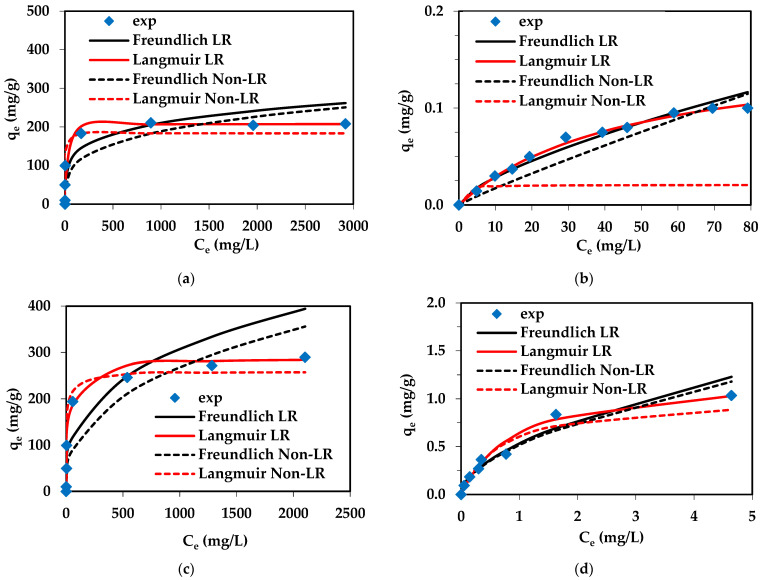
Experimental points and fitting of the Langmuir and Freundlich isotherms for Pd(II) (**a**,**c**) and Cu(II) (**b**,**d**) ion adsorption on the Diaion™ CR20 (**a**,**b**) and Lewatit^®^ VP OC 1065 (**c**,**d**).

- The Freundlich model [83,84]:

(21) $$\mathrm{The}\;\mathrm{linear}\;\mathrm{form}\;\;log\;q_{e}=log\;k_{F}+\frac{1}{n}log\;C_{e}$$

(22) $$\mathrm{The}\;\mathrm{non}-\mathrm{linear}\;\mathrm{form}\;{\;q}_{e}=k_{F}C_{e}^{1/n}$$

where *q*_e_—the amount of Pd(II) ions adsorbed per unit mass of ion exchanger (mg/g), *C*_e_—the equilibrium concentration of the solution (mg/L), *Q_0_*—the monolayer adsorption capacity (mg/g), *k_L_*—the Langmuir constant (related to the free energy of adsorption) (L/mg), *k*_F_ (mg^1−1/*n*^ L^1/*n*^/g), and 1/*n*—the Freundlich constants associated with the adsorption capacity of an adsorbent and the surface heterogeneity. The adsorption isotherm reflects the relationship between the amount of adsorbate adsorbed at a constant temperature and its concentration at equilibrium. The Langmuir isotherm model assumes that the adsorbent surface is covered with a single layer of adsorbate, whereas the Freundlich model is used to describe the multilayer adsorption on the heterogeneous adsorbent surface. In the Langmuir model, the adsorption sites are homogeneous, energetically equivalent, and without interactions between the adsorbed molecules [84,85]. The calculated values of the adsorption isotherm parameters at ambient temperatures are summarized in Table 6 for Pd(II) and in Table S3 for Cu(II), whereas Figure 12 shows the fitting of the isotherms to the adsorption data. 

The ion exchangers show different maximum adsorption capacities towards Pd(II) and Cu(II). Lewatit^®^ VP OC 1065 exhibited better efficiency for the Pd(II) adsorption compared to Diaion™ CR20, and the maximum values of adsorption capacities determined experimentally (*q_e exp_*) were found to be 289.68 mg/g and 208.2 mg/g, respectively. Both resins showed a slight capability of Cu(II) adsorption because the values of experimental adsorption capacities were small, up to 1.04 mg/g. The comparison of the obtained isotherms parameters based on the Langmuir and Freundlich isotherm models showed that the Pd(II) adsorption on both ion exchangers could be well described by the Langmuir model. This fact could be proven by the high values of the correlation coefficients obtained for the Langmuir model (*R*^2^ in the range 0.988–1.000—LR or 0.918–0.964—**Non—LR**) which are higher than those for the Freundlich model (*R*^2^ in the range 0.776–0.860 LR and **Non—LR**) and by the good agreement between the experimental and the calculated adsorption capacities, e.g., *q_e exp_* = 208.20 mg/g; *Q*_0_ = 207.59 mg/g for Diaion™ CR20 and *q_e exp_* = 289.68 mg/g; *Q*_0_ = 288.13 mg/g for Pd(II). The parameters 1/*n* obtained from the Freundlich model for Pd(II) were in the range of 0.207–0.383 for Pd(II), indicating that the adsorption of Pd(II) was favorable because the value of 1/*n* < 1. In the case of Cu(II) adsorption, the 1/*n* parameter was larger compared to Pd(II), being in the range of 0.541–0.916, also indicating a favorable adsorption, but smaller than in the case of Pd(II). In the case of Cu(II) adsorption on Lewatit^®^ VP OC 1065, the determination coefficients obtained from the linear (non-linear) regression were similar for both isotherm models: Langmuir *R*^2^ = 0.972 (0.952) and Freundlich *R*^2^ = 0.969 (0.946). In this case, one of the isotherm models that could be applied to describe the data could not be precisely specified. For Cu(II) adsorption on Diaion™ CR20, the determination coefficients were higher for the Freundlich model—*R*^2^ = 0.825 (0.823) compared to the Langmuir one. Taking into account the experimental maximum adsorption capacities towards Pd(II), Cu(II), Ni(II), and Zn(II), the following series could be found: Lewatit^®^ VP OC 1065: Pd(II) (289.68 mg/g) > Zn(II) (49.76 mg/g) = Ni(II) (49.76 mg/g) >> Cu(II) (1.04 mg/g); Diaion™ CR20: Pd(II) (208.20 mg/g) > Zn(II) (49.78 mg/g) ≈ Ni(II) (49.74 mg/g) >> Cu(II) (0.11 mg/g).

### 2.5. Column Studies

**Figure 13:**
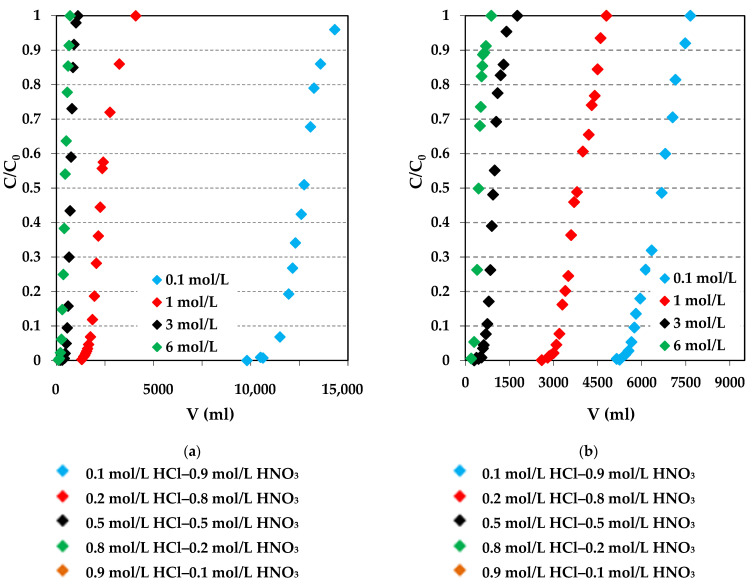

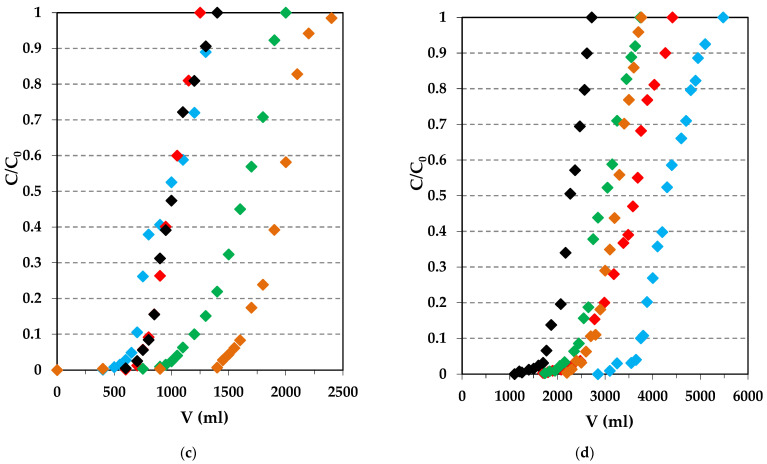
Comparison of the breakthrough curves of Pd(II) ion adsorption on Lewatit^®^ VP OC 1065 (**a**,**c**) and Diaion™ CR20 (**b**,**d**) from the chloride 0.1–6 mol/L HCl—100 mg Pd(II)/L (**a**,**b**) and the chloride-nitrate(V) solutions 0.1–0.9 mol/L HCl—0.9–0.1 mol/L HNO_3_—100 mg Pd(II)/L (**c**,**d**).

To design a column adsorption process, it is necessary to determine the breakthrough curve shape, the working ion exchange capacity of the adsorbent for the selected adsorbate under the given operating conditions. Therefore, the adsorption of Pd(II) and Cu(II) was performed using the column method described in the Experimental section, and the breakthrough curves are presented in Figure 13 for Pd(II) and Figure S4 for Cu(II). In the presented studies, the adsorption breakthrough curves were obtained at different acid concentrations (0.1–6 mol/L) and types (HCl, HCl-HNO_3_) at a certain flow rate (0.4 mL/min), with the bed volume being 10 mL and an initial concentration of 100 mg/L. The breakthrough curves were plotted, taking into account the quotient of Pd(II) or Cu(II) ion concentration in the eluate (*C*) to its initial concentration of the solution put into the column (*C*_0_) in the function of volume of the effluent collected from the column. The breakthrough points of the curves were determined when the Pd(II) or Cu(II) ion concentration reached 0.01 *C*_0_, whereas the effluent volume at *C*/*C*_0_ = 0.5 was used for the working ion exchange capacity calculation. When the effluent concentration became equal to the influent concentration, the saturation point was reached. As can be seen in Figure 13 and Figure S4, all breakthrough curves obtained for Pd(II) and Cu(II) on both ion exchangers followed the characteristic S-shaped profile. Moreover, the breakthrough points were observed at different effluent volumes depending on the acid types and concentrations. The breakthrough curves obtained from the chloride solutions were transferred to the higher effluent volume when the HCl concentration decreased from 6 to 0.1 mol/L. This tendency was observed for Pd(II) during adsorption on Lewatit^®^ VP OC 1065 and Diaion™ CR20. In the chloride-nitrate(V) solutions, the breakthrough curves obtained for the Pd(II) adsorption on Lewatit^®^ VP OC 1065 also moved to the larger effluent volume when the HCl concentration increased and the HNO_3_ concentration decreased. For the Pd(II) adsorption on Diaion™ CR20, this tendency was not maintained. Based on the calculated dynamic parameters collected in Table 7, it can be concluded that the working ion exchange capacities (*C_w_*) are strictly dependent on the acid concentration. The highest value of *C_w_* was obtained for the Pd(II)–Lewatit^®^ VP OC 1065 system (*C_w_* = 0.1050 g/mL, 0.1 mol/L HCl), which was almost twice as high as that for Diaion™ CR20 (*C_w_* = 0.0545 g/mL, 0.1 mol/L HCl). The capacities values decreased significantly with the increase in HCl concentration, and for 6 mol/L HCl being only 0.001 g/mL (Lewatit^®^ VP OC 1065) and 0.002 g/mL (Diaion™ CR20). The capacity reduction reached 99 or 96%. As the concentration of HCl increased, the concentration of Cl^−^ ions also increased, allowing for competition with chloroanionic species of palladium and resulting in a decrease in the working ion exchange capacities [86,87]. In the chloride-nitrate(V) solutions, the *C_w_* values increased with the HCl increase and HNO_3_ decrease, whereas, after exceeding the concentration of 0.5/0.5, they increased. The weight and bed distribution coefficients were also the largest in the 0.1 mol/L HCl for the Pd(II)–Lewatit^®^ VP OC 1065 system (*D_w_* = 3821.7 mL/g and *D_b_* = 1273.4). For the same HCl concentration and the Pd(II)–Diaion™ CR20 system, the values of the weight and bed distribution coefficients were much smaller (*D_w_* = 2077.3 mL/g and *D_b_* = 668.9), and the tendency of values to change was the same as in the case of the working ion exchange capacities, also due to the competitive effect. It was found that Lewatit^®^ VP OC 1065 showed a better Pd(II) adsorption ability from the diluted acidic solutions, whereas Diaion™ CR20 was more efficient for Pd(II) ion removal from the more concentrated acidic solutions. Also, a greater Diaion™ CR20 ability for Pd(II) compared to Lewatit^®^ VP OC 1065 was proven in the chloride-nitrate(V) solutions.

According to the results obtained during the Cu(II) adsorption on the Diaion™ CR20 and Lewatit^®^ VP OC 1065 ion exchangers, these ion exchangers sorbed Cu(II) to a small extent, and the working ion exchange capacities were in the range from 0.0001 to 0.0002 g/mL for the chloride solutions and from 0.0002 to 0.0003 g/mL for the chloride-nitrate(V) ones. 

The weight and bed distribution coefficients were very small, being in the range between 3.3 to 19.8 mL/g and 1.1 to 6.6. The selectivity series of both ion exchangers for Pd(II) and Cu(II) were the following:

- 0.1 mol/L HCl: Pd(II)—Lewatit^®^ VP OC 1065 > Pd(II)—Diaion™ CR20 >> Cu(II)—Lewatit^®^ VP OC 1065 > Cu(II)—Diaion™ CR20;
- 1–6 mol/L HCl: Pd(II)—Diaion™ CR20 > Pd(II)—Lewatit^®^ VP OC 1065 >> Cu(II)—Lewatit^®^ VP OC 1065 ≥ Cu(II)—Diaion™ CR20;
- 0.1–0.9 mol/L HCl—0.9–0.1 mol/L HNO_3_: Pd(II)—Diaion™ CR20 > Pd(II)—Lewatit^®^ VP OC 1065 > Cu(II)—Lewatit^®^ VP OC 1065 ≈ Cu(II)—Diaion™ CR20.

### 2.6. Desorption Studies

**Figure 14:**
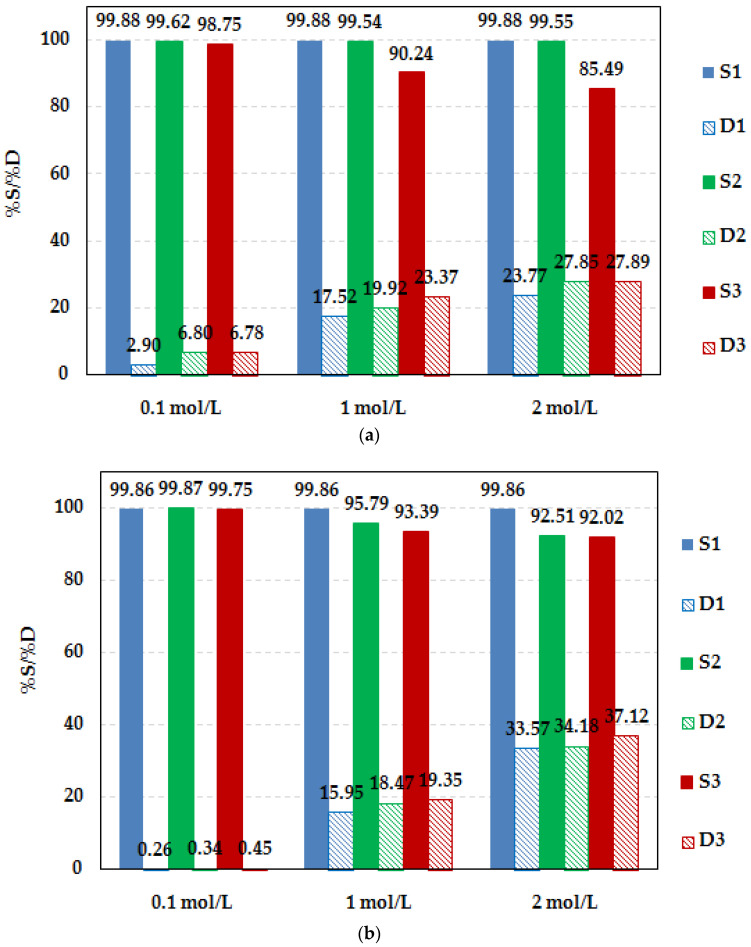
Comparison of the adsorption (%*S*) and desorption (%*D*) efficiency of Pd(II) ions on/from (**a**) Diaion™ CR20, (**b**) Lewatit^®^ VP OC 1065 ion exchangers in three adsorption–desorption cycles using ammonium hydroxide solutions.

Desorption tests of the retained metal(II) ions on the Lewatit^®^ VP OC 1065 and Diaion™ CR20 ion exchangers were carried out to verify their regenerability. The metal desorption step and recycling of the ion exchange resin are key to the implementation of the ion exchange system. The previously published data show that different systems have been tested for the recovery of metals loaded on the resin, including selected acids at different concentrations [86]. Based on the literature review [10,86,87], different chemical reagents such as HNO_3_, HCl, NH_3_·H_2_O, NaOH, and H_2_SO_4_ with 0.1, 1, and 2 mol/L concentrations were selected. The results are presented in Table 8 and Figure 14 for Pd(II). It was found that the desorption efficiency is dependent on the type of desorption solution, its concentration, and the ion exchanger used in the adsorption study. The desorption of Pd(II) ions from Diaion™ CR20 was not practically observed using the nitric(V) and sulfuric(VI) acids due to its very low desorption efficiency, which was in the ranges of 0.1–0.91% (HNO_3_) and 0.22–0.47% (H_2_SO_4_). A very small desorption yield was also obtained with NaOH solutions (%*D*_1,2,3_ = 0.47–1.54%). A slightly greater desorption efficiency was obtained with HCl solutions, and in this case, the %*D* increased with the increasing HCl concentration.

For 2 mol/L HCl, the desorption yield was equal to 14.73%. The best eluent for the Pd(II) desorption from Diaion™ CR20 was the NH_3_·H_2_O solution at 2 mol/L concentration (%*D*_1,2,3_ = 23.77–27.89%). The adsorption yield was still very large after three cycles of adsorption–desorption, and a slight decrease in the adsorption capacities was observed in the subsequent cycles. A similar capacity decrease has also been observed by other researchers [27,42,44]. 

In the Pd(II)—Lewatit^®^ VP OC 1065 system, the desorption efficiency was slightly greater compared to the Pd(II)—Diaion™ CR20 system using acidic solutions such as HNO_3_ (%*D*_1,2,3_ = 0.17–16.31%), H_2_SO_4_ (%*D*_1,2,3_ = 0.53–9.66%), and HCl (%*D*_1,2,3_ = 0.16–17.43%), and it increased with the increasing concentration of eluting agents. Using the NaOH solution for the desorption of Pd(II) from Lewatit^®^ VP OC 1065, the desorption was not satisfactory (%*D*_1,2,3_ = 0.13–2.15%), and the desorption efficiency was at a similar level to that in the Pd(II)—Diaion™ CR20 system. In all cases, the concentration of 2 mol/L allowed us to obtain a larger Pd(II) desorption yield compared to the 0.1 and 1 mol/L solutions, and NH_4_OH was found to be the best eluting agent (Figure 14b). The low desorption efficiency indicates that the interactions between the Pd(II) ions and the ion exchanger were rather strong.

For comparison, desorption studies of Cu(II) ions from Diaion™ CR20 were carried out (Figure S5). In this case, the desorption yield was much larger compared to Pd(II). In most cases, the desorption efficiency was the greatest using the eluting agents of the smallest concentration, which was 0.1 mol/L. Using the acidic solutions, most adsorbed Cu(II) ions could be effectively desorbed from Diaion™ CR20, and in this case, the acid concentration affected the desorption yield slightly (insignificant changes in %*D* were observed), e.g., for HNO_3_—%*D*_1_ = 96.12% (0.1 mol/L), %*D*_1_ = 94.55% (1 mol/L), %*D*_1_ = 95.58% (2 mol/L) whereas for HCl—%*D*_1_ = 88.72% (0.1 mol/L), %*D*_1_ = 84.26% (1 mol/L), %*D*_1_ = 86.63% (2 mol/L) and for H_2_SO_4_—%*D*_1_ = 90.59% (0.1 mol/L), %*D*_1_ = 91.55% (1 mol/L), and %*D*_1_ = 88.84% (2 mol/L). In the case of the NaOH and NH_3_·H_2_O solutions, a significant effect of desorption agent concentration on the desorption yield was observed. For the 1 and 2 mol/L concentrations of basic solutions, the %*D* values were small, in the range of 1–7%. The largest desorption was found using 0.1 mL/L NH_3_·H_2_O (%*D*_1_ = 99.58%), whereas for 0.1 mol/L NaOH %*D*_1_, it was equal to 86.41%. A moderate reduction in the ion exchangers’ capacities was observed in the subsequent cycles of adsorption.

### 2.7. Metal Adsorption from the Bi-Component Solutions

**Figure 15:**
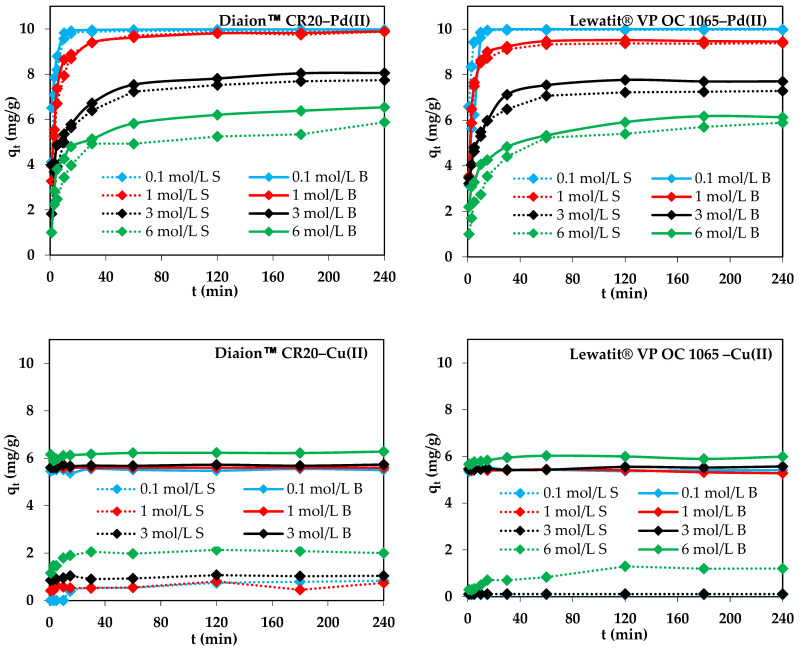
Effects of simultaneous presence of Pd(II) and Cu(II) ions in the solutions on their sorption yield on the Diaion™ CR20 and Lewatit^®^ VP OC 1065 ion exchangers from the S (single) and B (bi-component) solutions.

In order to evaluate the ion exchangers’ selectivity/affinity for Pd(II) and Cu(II), the bi-component solutions were prepared with an initial metal concentration of 100 mg/L (0.1–6 mol/L HCl—100 mg Pd(II)/L–100 mg Cu(II)/L). The comparison of the Pd(II) and Cu(II) ion adsorption yield obtained for Lewatit^®^ VP OC 1065 and Diaion™ CR20 in the bi-component solutions with that obtained using the single-component solutions is presented in Figure 15. The effect of the simultaneous presence of the metal Pd(II) and Cu(II) ions on their removal performance on ion exchangers was determined by calculating the ratio of adsorption capacity (*R_q_*_,*i*_) [88,89]:

(22) $$R_{q,i}=\frac{q_{2,i}}{q_{1,i}}$$

where *q*_2,*i*_—the adsorption capacity of metal *i* in the bi-component solution and *q*_1,*i*_—the adsorption capacity of metal *i* in the single-component solution under the same operating condition.

As follows from the literature [88], according to the values of *R_q_*_,*i*_, the presence of pollutants in multi-component systems could improve, reduce, or not change the adsorption efficiency: (a) *R_q_*_,*i*_ > 1: synergistic adsorption is observed (the presence of other pollutants in the multi-component systems improves the adsorption of pollutant *i*); (b) *R_q_*_,*i*_ = 0: there is no effect of the presence of other pollutants in the multi-component systems on the adsorption of pollutant *i*); and (c) *R_q_*_,*i*_ < 0: antagonistic adsorption is observed (the presence of other pollutants in the multi-component systems reduces the adsorption of pollutant *i*). By comparing the adsorption efficiency in the single- and bi-component solutions, it was observed that the simultaneous presence of Pd(II) and Cu(II) ions in the solution slightly increased the Pd(II) or significantly increased Cu(II) adsorption in the presence of second metal ions (e.g., for Diaion™ CR20: *q*_1,*Pd(II)*_ = 9.91 mg/g, *q*_2,*Pd(II)*_ = 9.98 mg/g and *q*_1,*Cu(II)*_ = 0.83 mg/g, *q*_2,*Cu(II)*_ = 5.50 mg/g, 0.1 mol/L HCl). The same tendency was observed in the systems with different HCl concentrations (e.g., for Diaion™ CR20: *q*_1,*Pd(II)*_ = 5.88 mg/g, *q*_2,*Pd(II)*_ = 6.54 mg/g and *q*_1,*Cu(II)*_ = 2.00 mg/g, *q*_2,*Cu(II)*_ = 6.28 mg/g, 6 mol/L HCl). The value of the ratio of the adsorption capacity for Pd(II) is close to 1 or negligibly higher than 1 (*R_q_*_,*Pd(II)*_ in the range from 1 to 1.11 for the system with Diaion™ CR20 and from 1 to 1.06 for the system with Lewatit^®^ VP OC 1065), thereby confirming no effect or a negligible synergetic adsorption effect of Pd(II) in the presence of Cu(II) in the bi-component solutions. On the other hand, in the case of Cu(II), the *R_q_*_,*Cu(II)*_ takes significantly greater than 1 values, confirming the synergetic adsorption in the bi-component solutions. The fact that the adsorption amount for Cu(II) increases significantly in the presence of Pd(II), while for Pd(II), it remains at a practically similar level in the presence of Cu(II), indicates stronger interactions between Cu(II) and the surface of the ion exchanger compared with those involved in the Pd(II) adsorption in the binary system. As follows from the literature, the adsorption of Cu(II) from the single- and bi-component solutions (Pd(II)–Cu(II)) on bentonite depends largely on the nature of bentonite and the presence of Pd(II) ions. The presence of Pd(II) suppresses the copper(II) adsorption (the values of %*R* were reduced from 69% to 23% for N-Bent(G) and from 61% to 31% for N-Bent(D)) [90]. On the other hand, the Pd(II) adsorption on the pyromellitic acid-modified UiO–66–NH_2_ from the multi-component solutions containing Cu(II), Mn(II), Zn(II), Pb(II), and Pd(II) of 100 mg/L concentration (10 mg of sorbent, 15 mL of solution, pH 2.0, time 24 h) showed a greater ability for Pd(II) adsorption than for Cu(II) (the distribution coefficient was 36,670.9 mL/g for Pd(II) and 9.8 mL/g for Cu(II)) [91]. 

## 3. Materials and Methods

### 3.1. Materials

In the studies, the following reagents and materials were used: palladium chloride (PdCl_2_), copper(II) chloride dihydrate salt (CuCl_2_·2H_2_O), hydrochloric acid (HCl), nitric(V) acid (HNO_3_), sulfuric(VI) acid (H_2_SO_4_), sodium hydroxide (NaOH), ammonia aqueous solution (NH_3_∙H_2_O), and ion exchangers: Lewatit^®^ VP OC 1065 and Diaion™ CR20. Salts, acids, and bases of analytical grade used in the studies were purchased from Sigma Aldrich (St. Louis, MO, USA) or Chempur (Piekary Śląskie, Poland). Diaion™ CR20 was supplied by Mitsubishi Chemical Industries (Tokyo, Japan) and Lewatit^®^ VP OC 1065 by Lanxess (Cologne, Germany). 

Palladium(II) and copper(II) stock solutions of 10 000 mg/L concentrations were prepared by dissolving appropriate quantities of PdCl_2_ or CuCl_2_·2H_2_O salts in 0.1 mol/L HCl. Working solutions of the appropriate concentration (*C*_0_, mg/L) were prepared from the stock solutions by dilution to obtain the desired concentrations of metal ions and by the addition of HCl or HCl—HNO_3_ to obtain different acid concentrations. The chloride: 0.1–6.0 mol/L HCl or chloride-nitrate(V) 0.1–0.9 mol/L HCl—0.9–0.1 mol/L HNO_3_ solutions containing Pd(II) or Cu(II) ions were obtained and designated in the paper as:

- Chloride solutions (systems S1–S4):

0.1 mol/L HCl (S1), 1 mol/L HCl (S2), 3 mol/L HCl (S3), 6 mol/L HCl (S4),

- Chloride-nitrate(V) solutions (systems S5–S9):

0.1 mol/L HCl–0.9 mol/L HNO_3_ (S5), 0.2 mol/L HCl–0.8 mol/L HNO_3_ (S6), 0.5 mol/L HCl–0.5 mol/L HNO_3_ (S7), 0.8 mol/L HCl–0.2 mol/L HNO_3_ (S8), 0.9 mol/L HCl–0.1 mol/L HNO_3_ (S9).

**Figure 16:**
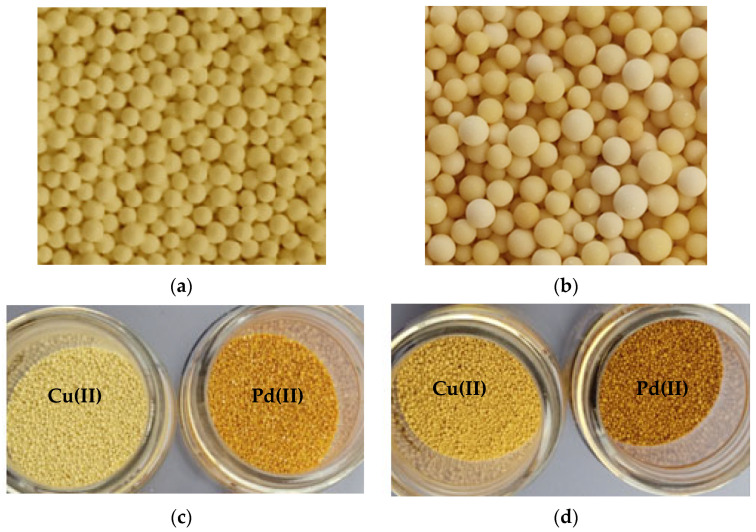
Diaion™ CR20 (**a**,**c**) and Lewatit^®^ VP OC 1065 (**b**,**d**) ion exchange resins beads before the adsorption (**a**,**b**) (magnification 5×) and after the Cu(II) and Pd(II) adsorption (**c**,**d**) (magnification 2.5×).

Two ion exchange resins with the commercial names Lewatit^®^ VP OC 1065 and Diaion™ CR20 were selected and used in the investigations (Figure 16). The ion exchange resins were purified before use by rinsing in water and transformed from the free base form to the chloride one using hydrochloric acid in a 1 mol/L concentration. The ion exchanger, after being swollen in the aqueous solutions, was used in the column studies or dried at room temperature, and in this form, it was applied for the static adsorption tests. The general description of these materials is as follows:

- Lewatit^®^ VP OC 1065—a polystyrene–divinylbenzene macroporous matrix with the primary amine functional groups, weakly basic, bead size of 1.25–0.315 mm, total exchange capacity 2.2 eq/L, water content 65–70%, ionic form as shipped free base, and appearance of opaque beads.
- Diaion™ CR20—a polystyrene–divinylbenzene macroporous matrix with polyamine amine functional groups, chelating ion exchanger, bead size of 1.2–0.300 mm, total exchange capacity toward copper min. 0.4 mmol/mL, water content 50–60%, ionic form as shipped free base, and appearance of opaque beads.

The samples of Lewatit^®^ VP OC 1065 and Diaion™ CR20 ion exchangers before the adsorption process were tested using the CHNS analyzer (Elemental Analyser Vario EL III), which enables simultaneous determination of the percentage contents of carbon, hydrogen, nitrogen, and sulfur. The CHNS analysis procedure includes the following steps: (a) weighing the samples using the Sartorius M2P analytical microbalance with an accuracy of 0.001 mg, (b) catalytic combustion of the sample at 1200 °C (resulting in the release of N_2_, CO_2_, and H_2_O), (c) separation of gases on the adsorption columns, and (d) detection based on the difference in thermal conductivity using the katharometer. The combustion processes were repeated three times for all samples.

The surface properties of the Lewatit^®^ VP OC 1065 and Diaion™ CR20 ion exchangers were determined using a Quantachrome Autosorb iQ Analyzer (Quantachrome, Graz, Austria) at 77 K using nitrogen gas. The samples were degassed at *T* = 343 K for over 12 h. The specific surface area (*S_BET_*), the total pore volumes (*V_tot_*), and the average pore diameter (*D*) were calculated using the adsorption data. The specific surface area (*S_BET_*) was calculated using the Brunauer–Emmett–Teller (BET) method, whereas the Barrett–Joyner–Halenda (BJH) one was applied in the pore diameter and volume analyses. 

The pH-drift method [22] was applied to determine the pH of the point of zero charge (*pH_PZC_*) of the ion exchangers. The 0.01 mol/L KNO_3_ solutions of *V* = 50 mL in volume of different initial pH_i_ (1–12) (prepared using HCl and NaOH of 0.1 mol/L or 2 mol/L) were shaken under an agitation speed of *V_as_* = 180 rpm and an amplitude of *A* = 8, with the mass of the ion exchange resins equal to *m* = 0.5 ± 0.0005 g. The final pH_f_ was measured after time *t* = 24 h using a pH meter CP-411 (Elmetron, Zabrze, Poland). The pH changes were calculated (∆pH = pH_i_ − pH_f_) by subtracting the final pH from the initial one. The *pH_PZC_* is the point where pH_i_ − pH_f_ = 0. 

The Attenuated Total Reflectance–Fourier Transform Infrared Spectroscopy (ATR-FTIR) analysis was used to study the ion exchangers’ infrared spectra under discussion before and after the Pd(II) and Cu(II) ion adsorption, applying the Cary 630 FTIR analyzer (Agilent Technologies, Santa Clara, CA, USA) and the Agilent MicroLab PC software (version: B.04). The FTIR spectra were recorded in the frequency range of 4000–500 cm^−1^ to determine the ion exchangers’ functional groups. The band intensities were expressed in absorbance. For the post-collection analysis of the spectra, the Agilent Resolutions Pro software was applied.

A detailed characterization of the ion exchange resins is presented in Section 2. 

### 3.2. Batch Adsorption Method—Experimental Conditions

In the batch method, conical flasks of 100 mL volume closed with silicone stoppers were used. Firstly, 50 mL of solution containing Pd(II) or Cu(II) was added into the equal quantities of Lewatit^®^ VP OC 1065 or Diaion™ CR20 (*m* = 0.5 ± 0.0005 g). The flasks were placed in an Elphin+ 357 mechanical shaker (Lubawa, Poland). The batch adsorption experiments were conducted by shaking the Erlenmeyer flasks at a constant speed and amplitude (*A* = 8) at ambient temperature and under atmospheric pressure. The ion exchanger beads were separated from the solution after a proper period of time by filtration. Next, the final Pd(II) or Cu(II) metal ion concentration was analyzed by the Atomic Absorption Spectrometry Method (AAS) (spectrometer Varian AA240FS, Varian, Mulgrave, Australia) using standard solutions and the calibration curve. The parameters in the AAS metal content determination were the following: wavelength—232 nm for Cu(II) and 247,6 nm for Pd(II); slot width—2 nm for Cu(II) and 0.2 for Pd(II); ratio of air to acetylene: 13.5:2 L/min for both metal ions; and lamp current—4 mA for Cu(II) and 10 mA for Pd(II). All the experiments were performed in triplicate, and the averaged values were the final ones. Kinetic, equilibrium, and desorption studies were carried out and several conditions were studied, including the effects of phase contact time and the acid concentration, the agitation speed, the initial concentration of M(II) (Cu(II) or Pd(II)), the temperature, and the bead size. The experimental conditions are presented in Table 9. The adsorption/desorption studies of M(II) using the static method were carried out in triplicate, and the graphs were presented as the arithmetic mean of the results. In all cases, the standard deviation did not exceed 3.5%.

To examine the ion exchangers’ selectivity/affinity for Pd(II) and Cu(II), the adsorption was performed from the bi-component solutions of different HCl concentrations (0.1–6 mol/L HCl—100 mg Pd(II)/L–100 mg Cu(II)/L), and the ratio of adsorption capacity (*R_q_*_,*i*_) was calculated.

### 3.3. Column Adsorption Procedure and Calculation

The column studies were carried out in ion exchange glass columns 1 cm in internal diameter and 25 cm in height, which were packed with 10 mL of swollen ion exchanger. The solution with the initial Pd(II) or Cu(II) concentration equal to 100 mg/L was passed through the prepared bed at a flow rate of 0.4 mL/min at room temperature (293 ± 2 K). The eluate was collected into the defined volume fractions (5–500 mL), and AAS spectrometry was used to determine the contents of the tested metal ions in the effluent. The column process was repeated until a saturation point was obtained, which means that the concentration of M(II) ions in the effluent was equal to the initial concentration of the solution introduced into the column (100 mg/mL). Then, the breakthrough curves were obtained and the dynamic parameters, such as the working ion exchange capacity (*C_w_*), weight (*D_w_*), and bed distribution coefficients (*D_b_*), were calculated using the following formulae:

(23) $$C_{w}=\frac{U_{p}\cdot C_{0}}{V_{j}}$$

(24) $$D_{w}=\frac{U^{\prime \prime }-U_{0}-V}{m_{j}}$$

(25) $$D_{b}=D_{w}\cdot \;d_{z}$$

where: *U_p_*—the volume of eluate to the breakthrough point (L), *C*_0_—the initial M(II) concentration (g/L), *V_j_*—the volume of the ion exchange in the packed column, *U*″—the eluate volume at *C/C*_0_ = 0.5 (mL), *U*_0_—the dead column volume (*U*_0_ = 2 mL), *V*—the free volume of the ion exchange bed (about 0.4 bed volume) (mL), *m_j_*—the mass of dry ion exchange resin in the column (g); and *d_z_*—the ion exchange density (determined experimentally) (g/mL).

### 3.4. Desorption Studies

The desorption experiments were conducted by means of the static method. The previously adsorbed Pd(II) or Cu(II) ions on the ion exchangers (0.5 g) were transferred to a flask containing 50 cm^3^ of a desorbing agent such as HNO_3_, HCl, NH_3_∙H_2_O, NaOH, or H_2_SO_4_. The effects of eluting agent concentrations (0.1, 1, 2 mol/L) on the desorption efficiency were investigated. The mixture was shaken under the experimental conditions of *V_as_* = 180 rpm, *A* = 8, *T* = 293 K for 240 min, and then the phases were separated and the concentration of M(II) ions in the aqueous solution after desorption was determined by means of the atomic absorption spectroscopy (AAS) method. To explore the potential reusability of ion exchangers, the 3 cycles of adsorption and desorption were examined using the best eluting agent selected during the first desorption step.

## 4. Conclusions

The objective of this paper was to conduct an evaluation of the suitability of the polystyrene–divinylbenzene macroporous ion exchangers, Lewatit^®^ VP OC 1065 and Diaion™ CR20, as potential sorbents for the removal of Pd(II) and Cu(II) ions from single and binary aqueous solutions. The characterization of the ion exchangers, as well as the effects of HCl concentration, initial metal ion concentration, agitation speed, ion exchanger bead size, and temperature, were considered. The conditions of 0.1 mol/L HCl, 180 rpm, and bead size fraction (0.3–1.2 mm) for Diaion™ CR20 and (0.315–1.25 mm) for Lewatit^®^ VP OC 1065, as well as a temperature of 293 K, are the optimal conditions for the Pd(II) adsorption process. In the case of Cu(II), the optimal conditions ensuring the largest uptake are 6 mol/L HCl, 180 rpm, f4 (0.385 mm ≤ f4 < 0.43 mm), and the ambient temperature, but in this case, the adsorption was very low (Pd(II) >> Zn(II) ≈ Ni(II) >> Cu(II)). Lewatit^®^ VP OC 1065 is characterized by better kinetic and Pd(II) adsorption ability than Diaion™ CR20, which means that more Pd(II) ions are removed with a better efficiency in a shorter period of time from the diluted acidic solutions. Moreover, the ability of this ion exchanger was also confirmed in the column studies. Therefore, Lewatit^®^ VP OC 1065 will be a potential sorbent for wastewater treatment containing heavy metals on a larger scale, and could be taken into account during the development of effective methods for their elimination.

## Figures and Tables

**Table 1:** Physicochemical properties of Lewatit^®^ VP OC 1065 and Diaion™ CR20 resins [22,23,24,25,26].

Properties	Lewatit^®^ VP OC 1065	Diaion™ CR20
Type	weakly basic anion exchanger	chelating ion exchanger
Matrix	polystyrene–divinylbenzene (8–10% DVB) [26]	polystyrene–divinylbenzene
Structure	macroporous	macroporous
Functional groups	primary amine (benzylamine) 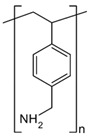	polyamine 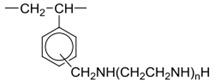
Ionic form as shipped	free base (FB)	free base (FB)
Appearance	opaque beads	opaque beads
Bead size	0.315–1.25 mm (>90%)	0.300–1.2 mm (min. 95%) particle size distribution on 1.18 mm (max. 5%), mean particle size 0.57 mm
Fines	max. vol. 3% (<0.315 mm)	max. vol. 1% (<0.300 mm)
Effective size	0.47–0.57 mm	0.4 mm
Uniformity coefficient	max. 1.8	1.6
Total capacity	min. 2.2 eq/L	1.4 eq/L min. 0.4 mmol/mL for Cu
Operating temperature	max. 100 °C	max. 100 °C
Effective pH range	-	4 *–10 ** [24], 2–6 [25]
Shipping density	630–710 g/L	640 g/L
Total swelling, FB to Cl^−^	-	10%
Application	-selective adsorption of compounds containing acidic groups, -decolorization of sugar starch and protein solutions, -adsorption of atmospheric CO_2_ and aldehydes	-chemical process separations, -metals removal and recovery from wastewater: the selectivity towards metal ions: Hg^2+^ > Fe^3+^ > Cu^2+^ > Zn^2+^ > Cd^2+^ > Ni^2+^ > Co^2+^ > Ag^+^ > Mn^2+^

- Data not available. * Some metal ions can be slightly adsorbed at a pH lower than 4. ** In an alkaline solution, ions can be precipitated as hydroxide.

**Table 2:** Comparison of the measurement results using the low-temperature N_2_ adsorption/desorption method for Lewatit^®^ VP OC 1065 and Diaion™ CR20 resins.

Ion Exchanger	Textural Parameters		
	*S_BET_* (m^2^/g)	*V_tot_* (mL/g)	*D* (nm)
Lewatit^®^ VP OC 1065	3.194	0.0285	35.74
	BET approx. 50 [22] BET ^1^ 26.2, ^2^ 11.9 [25]	approx. 0.27 [22] BJH ^1^ 0.26, ^2^ 0.08 [25]	Pore diameter: avg. 25 [22] Pore radius: BJH ^1^ 15.4; ^2^ 15.2 [25]
Diaion™ CR20	44.538	0.7668	68.87

^1^ 120 °C, 2 h in vacuum 30 mm Hg, ^2^ 120 °C, 24 h in vacuum 30 mm Hg.

**Table 3:** Effects of agitation speed on the Pd(II) adsorption on ion exchangers/sorbents of different types.

Sorbent	Types	Properties	Conditions	Optimal Agitation Speeds	Ref.
Dowex M4195	chelating resin	M: St-DVB, S: macroporous, FG: bis-picolylamine	systems: 0.1 mol/L HCl–1.0 mol/L NaCl, 2.0 mol/L HCl–1.0 mol/L NaCl, 1.0 mol/L NaCl, *m* = 0.5 ± 0.0005 g, *C*_0_ = 100 mg Pd(II)/L, *V* = 0.05 L, *V_as_* = 180 rpm, *A* = 8 and 350 rpm, *A* = 4, *T* = ambient, *t* = 1–720 min	180 rpm, *q_e_* increases with *V_a_* increases up to 360 min, the system reaches equilibrium after a shorter period of time at 350 rpm	[43]
Lewatit MonoPlus TP220	chelating resin	M: St-DVB, S: macroporous, FG: bis-picolylamine	systems: 0.1 mol/L HCl (S1), *m* = 0.5 ± 0.0005 g, *C*_0_ = 500 mg Pd(II)/L, *V* = 0.05 L, *V_as_* = 120, 150, 180 rpm, *A* = 8, *T* = ambient, *t* = 1–1440 min	180 rpm, *q_e_* sligtly increases with phase contact time at 120 rpm; for 150 and 180 rpm, *q_e_* increases are not evident	[44]
Lewatit MonoPlus SR7	strongly basic anion exchanger	M: polistyrene, S: macroporous, FG: quaternary ammonium	systems: 0.1 mol/L HCl (S1), 0.9 mol/L HCl–0.1 mol/L HNO_3_ (S9), *m* = 0.5 ± 0.0005 g, *C*_0_ = 100 mg Pd(II)/L, *V* = 0.05 L, *V_as_* = 120, 150, 180 rpm, *A* = 8, *T* = 298 ± 2 K, *t* = 1–1440 min	180 rpm, *q_e_* increases with *V_a_* increases up to 60–120 min, *q_e_* = 9.99 mg/g at 1440 min, increase in the total concentration of the Cl^−^ leads to the slowest kinetics	[55]
Dowex PSR2	strongly basic anion exchanger	M: St-DVB, S: micro- (PSR2) and macro- (PSR3) porous FG: tri-n-butylamine	systems: 0.1 mol/L HCl (S1), 0.9 mol/L HCl–0.1 mol/L HNO_3_ (S9), *m* = 0.5 ± 0.0005 g, *C*_0_ = 500 mg Pd(II)/L, *V* = 0.05 L, *V_as_* = 120, 150, 180 rpm, *A* = 8, *T* = ambient, *t* = 1–240 min	180 rpm, *q_e_* changes negligibly, the highest *q_e_* changes are observed for Pd(II) adsorption on Dowex PSR2 from S9 (*q_e_* increases from 23.9 to 26.3 mg/g); in S9, *V_a_* increases up to 60 min for the 120 rpm for Dowex PSR3	[56]
Dowex PSR3					
Purolite A400TL	strongly basic anion exchanger	M: St-DVB, S: microporous FG: quaternary ammonium	systems: 0.1 mol/L HCl (S1), 0.9 mol/L HCl–0.1 mol/L HNO_3_ (S9), *m* = 0.5 ± 0.0005 g, *C*_0_ = 500 mg Pd(II)/L, *V* = 0.05 L, *V_as_* = 120, 150, 180 rpm, *A* = 8, *T* = ambient, *t* = 1–240 min, *S_b_* = 0.425–0.85 mm	180 rpm, *q_e_* increases with *V_a_* increases up to 60–120 min, then varies by less than 0.5% for S1 and 3% for S9; when *V_a_* increases, the time required to reach an equilibrium decreases from 60–120 min to 30 min (S1,S9)	[56]
Purolite A830	weakly basic anion exchanger	M: Pac-DVB, S: macroporous, FG: complex amine	systems: 0.1 mol/L HCl (S1), 0.9 mol/L HCl–0.1 mol/L HNO_3_ (S9), *m* = 0.5 ± 0.0005 g, *C*_0_ = 100 mg Pd(II)/L, *V* = 0.05 L, *V_as_* = 120, 150, 180 rpm, *A* = 8, *T* = 298 ± 2 K, *t* = 1–1440 min	180 rpm, *q_e_* increases with *V_a_* increases up to 60–120 min, *q_e_* = 9.7–10 mg/g; when *V_a_* increases, the system reaches equilibrium after a shorter period of time, i.e., after 15 (S1) and 120 min (S9)	[58]
Fe_3_O_4_-CSN	magnetic chitosan nanoparticles		*m* = 1.5 g/L of Fe_3_O_4_-CSN, *C*_0_ = 10 mg Pd(II)/L, *V* = 0.05 L, *V_a_*_s_ = 100–1400 rpm, *t* = 10 min, pH 6, *T* = 20 ± 1 °C	500 rpm, *q_e_* sligtly decreases at *V_a_* above 500 rpm	[59]
Lewatit AF5	resin without FG	M: carbonaceous, S: microporous, FG: none	systems: 0.1 mol/L HCl (S1), 0.9 mol/L HCl–0.1 mol/L HNO_3_ (S9), *m* = 0.5 ± 0.0005 g, *C*_0_ = 500 mg Pd(II)/L, V = 0.05 L, *V_as_* = 120,140,180 rpm, *A* = 8, *T* = ambient, *t* = 1–240 min	180 rpm, *q_e_* sligtly increases with *V_a_*, tendency is much more marked in S1 than S9 solutions at the beginning of the adsorption process	[60]

M—the matrix of ion exchanger, St-DVB—the styrene–divinylbenzene, PAc-DVB—the polyacrylic–divinylbenzene, S—the structure, FG—the functional groups, *q_e_*—the values of adsorption capacities.

**Table 4:** Kinetic models’ characteristics [74,75,76,77,78,79,80,81].

Kinetic Model	Non-Linear Equation			Linear Equation			Parameters	
The pseudo-first-order kinetic model, PFO	$q_{t}=q_{e}\left(1-e^{-k_{1}t}\right)$ (10)			$\log\left(q_{e}-q_{t}\right)=\log q_{e}-\frac{k_{1}}{2.303}t$ (11)			*k*_1_ = −2.303 × *slope* *q_e_* = 10*^intercept^*	
The pseudo-second-order kinetic model, PSO	$\;\;\;\;\;\;q_{t}=\frac{q_{e}^{2}k_{2}t}{q_{e}k_{2}t+1}$ (12)			$\frac{t}{q_{t}}=\frac{1}{k_{2}q_{e}^{2}}+\frac{1}{q_{e}}t$ (13)			*k*_2_ = *slope*^2^/*interceptq_e_* = 1/*slope*$h=k_{2}q_{e}^{2}\;\;\;\;\;\;\;\;(14)$	
The intraparticle diffusion model, IPD	*-*			$q_{t}=k_{i}t^{1/2}+C$ (15)			*k_i_ = slope* *C = intercept*	
Error analysis								
$MPSD=\sum_{i=1}^{n}{\left(\frac{q_{e\;exp}-q_{e\;cal}}{q_{e\;exp}}\right)}_{i}^{2}$		(16)	$R^{2}=1-\frac{{\sum \left(q_{e\;exp}-q_{e\;cal}\right)}^{2}}{{\sum \left(q_{e\;exp}-q_{e\;mean}\right)}^{2}}$		(17)	$R_{adj}^{2}=1-\left[\frac{\left(1-R^{2})(n-1\right)}{n-k-1}\right]$		(18)

*q_e_* and *q_t_* (mg/g)—the amounts of M(II) sorbed at the equilibrium and at any time *t*, *k*_1_ (1/min)—the rate constants determined from the PFO equation, *t*—the contact time, *k*_2_ (g/mg min)—the rate constants determined from the PSO equation, *h*—the initial adsorption rate, *k_i_* (mg/g min^0^.^5^)—the intraparticle diffusion rate constant, *C*—the Weber–Morris diffusion constant, *q_e mean_* (mg/g)—measured by means of *q_e exp_* values, *n*—the points number in the data sample, *k*—the number of independent regressors.

**Table 5:** Kinetic parameters for the Pd(II) adsorption on the ion exchangers from the 0.1–6 mol/L HCl—100 mg/L systems using the PSO model.

Parameters		Diaion™ CR20				Lewatit^®^ VP OC 1065			
		0.1 mol/L HCl	1 mol/L HCl	3 mol/L HCl	6 mol/L HCl	0.1 mol/L HCl	1 mol/L HCl	3 mol/L HCl	6 mol/L HCl
*q_e exp_* (mg/g)		9.90	9.90	7.70	5.90	9.97	9.38	7.29	5.89
**PSO** **LN**	*q_e cal_* (mg/g)	9.939	9.938	7.868	5.807	9.987	9.438	7.370	6.037
	*k*_2_ (g/mg min)	0.149	0.047	0.025	0.023	0.373	0.085	0.048	0.018
	*R* ^2^	1.000	1.000	1.000	0.995	1.000	1.000	1.000	0.999
	*h*	37.18	7.53	2.61	0.65	14.74	4.60	1.54	0.78
**PSO** **Non—LN**	*q_e cal_* (mg/g)	10.29	9.90	7.34	5.42	10.12	9.63	6.78	5.42
	*k*_2_ (g/mg min)	0.068	0.046	0.040	0.038	0.185	0.060	0.103	0.038
	*MPSD*	0.011	0.013	0.051	0.038	0.003	0.008	0.103	0.234
	*R* ^2^	0.974	0.991	0.966	0.977	0.976	0.986	0.873	0.942
	*R* ^2^ *adj*	0.966	0.989	0.956	0.970	0.969	0.982	0.837	0.925

**Table 6:** Isotherm parameters for the Pd(II) ion adsorption on the Diaion™ CR20 and Lewatit^®^ VP OC 1065 ion exchangers.

Model	Parameters	Diaion™ CR20	Lewatit^®^ VP OC 1065
		Pd(II)	Pd(II)
*q_e exp_* (mg/g)		208.20	289.68
**Linear regression**			
**Langmuir**	*Q*_0_ (mg/g)	207.59	288.13
	*k_L_* (L/mg)	0.190	0.032
	*R* ^2^	1.000	0.998
**Freundlich**	*k_F_* (mg^1−1/*n*^ L^1/*n*^/g)	50.35	29.84
	1/*n*	0.207	0.337
	*R* ^2^	0.776	0.830
**Non-linear regression**			
**Langmuir**	*Q*_0_ (mg/g)	183.48	258.85
	*k_L_* (L/mg)	1.286	0.087
	*MPSD*	0.719	0.215
	*R* ^2^	0.918	0.964
	*R* ^2^ *_adj_*	0.877	0.946
**Freundlich**	*k_F_* (mg^1−1/*n*^ L^1/*n*^/g)	29.73	18.95
	1/*n*	0.267	0.383
	*MPSD*	1.233	1.078
	*R* ^2^	0.857	0.860
	*R* ^2^ *_adj_*	0.785	0.790

**Table 8:** Comparison of the adsorption (%*S*) and desorption (%*D*) efficiency of Pd(II) ions on/from the Diaion™ CR20 and Lewatit^®^ VP OC 1065 ion exchangers in the three adsorption–desorption cycles.

Eluting Agent		0.1 M HCl—100 mg Pd(II)/L											
		Diaion™ CR20						Lewatit^®^ VP OC 1065					
		*%S*_1_	*%D_1_*	*%S*_2_	*%D*_2_	*%S*_3_	*%D*_3_	*%S*_1_	*%D*_1_	*%S*_2_	*%D*_2_	*%S*_3_	*%D*_3_
1	0.1 mol/L HNO_3_	99.88	0.04	99.85	0.04	99.72	0.35	99.86	0.18	99.67	0.19	99.64	0.17
2	1 mol/L HNO_3_	99.88	0.08	99.77	0.10	99.75	0.30	99.86	2.76	99.65	12.77	99.54	11.71
3	2 mol/L HNO_3_	99.88	0.47	99.70	0.40	99.71	0.91	99.86	4.10	99.36	14.60	98.50	16.31
4	0.1 mol/L HCl	99.88	0.00	99.78	0.06	99.75	0.10	99.86	0.16	99.83	0.20	99.74	0.18
5	1 mol/L HCl	99.88	0.82	99.70	1.23	99.81	1.53	99.86	3.13	99.74	3.59	99.05	4.82
6	2 mol/L HCl	99.88	7.72	99.88	8.73	99.78	9.74	99.86	7.98	99.71	13.38	99.37	14.73
7	0.1 mol/L NH_3_·H_2_O	99.88	2.90	99.62	6.80	98.75	6.78	99.86	0.26	99.87	0.34	99.75	0.45
8	1 mol/L NH_3_·H_2_O	99.88	17.52	99.54	19.92	90.24	23.37	99.86	15.95	95.79	18.47	93.39	19.35
9	2 mol/L NH_3_·H_2_O	99.88	23.77	99.55	27.85	85.49	27.89	99.86	33.57	92.51	34.18	92.02	37.12
		Figure 14a						Figure 14b					
10	0.1 mol/L NaOH	99.88	1.43	99.63	1.18	99.53	1.23	99.86	2.15	99.50	1.54	99.55	1.20
11	1 mol/L NaOH	99.88	1.52	99.59	1.13	99.49	1.48	99.86	0.76	99.84	0.50	99.82	0.45
12	2 mol/L NaOH	99.88	0.47	99.53	1.13	99.68	1.54	99.86	0.32	99.87	0.14	99.91	0.13
13	0.1 mol/L H_2_SO_4_	99.88	0.30	99.43	0.47	99.64	0.26	99.86	0.65	99.55	0.58	99.31	0.53
14	1 mol/L H_2_SO_4_	99.88	0.23	99.62	0.22	99.52	0.33	99.86	1.46	98.84	3.44	97.58	5.03
15	2 mol/L H_2_SO_4_	99.88	0.26	99.57	0.25	99.51	0.39	99.86	3.05	97.50	7.79	95.83	9.66

**Table 7:** Dynamic study parameters of Pd(II) and Cu(II) ion adsorption onto the Diaion™ CR20 and Lewatit^®^ VP OC 1065 ion exchangers.

System	Lewatit^®^ VP OC 1065			Diaion™ CR20		
	*C_w_* (g/mL)	*D_w_* (mL/g)	*D_b_*	*C_w_* (g/mL)	*D_w_* (mL/g)	*D_b_*
	**Pd(II)**					
0.1 mol/L HCl	0.1050	3821.7	1273.4	0.0545	2077.3	668.9
1 mol/L HCl	0.0140	694.5	231.4	0.0280	1184.5	381.4
3 mol/L HCl	0.0040	214.3	71.4	0.0050	296.3	95.4
6 mol/L HCl	0.0010	127.3	42.4	0.0020	137.9	44.4
0.1 mol/L HCl–0.9 mol/L HNO_3_	0.0050	292.3	97.4	0.0310	1327.3	427.4
0.2 mol/L HCl–0.8 mol/L HNO_3_	0.0070	298.3	99.4	0.0180	1122.4	361.4
0.5 mol/L HCl–0.5 mol/L HNO_3_	0.0070	298.3	99.4	0.0140	701.6	225.9
0.8 mol/L HCl–0.2 mol/L HNO_3_	0.0090	491.9	163.9	0.0184	928.3	298.9
0.9 mol/L HCl–0.1 mol/L HNO_3_	0.0140	584.9	194.9	0.0230	1007.5	324.4
	**Cu(II)**					
0.1 mol/L HCl	0.0002	16.2	5.4	0.0001	6.2	2.0
1 mol/L HCl	0.0002	4.8	1.6	0.0001	6.2	2.0
3 mol/L HCl	0.0002	4.2	1.4	0.0001	6.5	2.1
6 mol/L HCl	0.0001	19.8	6.6	0.0001	10.9	3.5
0.1 mol/L HCl–0.9 mol/L HNO_3_	0.0003	6.3	2.1	0.0003	6.6	2.1
0.2 mol/L HCl–0.8 mol/L HNO_3_	0.0002	3.3	1.1	0.0003	5.3	1.7
0.5 mol/L HCl–0.5 mol/L HNO_3_	0.0002	5.4	1.8	0.0002	5.2	1.7
0.8 mol/L HCl–0.2 mol/L HNO_3_	0.0002	4.8	1.6	0.0002	6.7	2.2
0.9 mol/L HCl–0.1 mol/L HNO_3_	0.0003	6.3	2.1	0.0002	5.2	1.7

**Table 9:** Experimental conditions applied in the kinetic, equilibrium, and desorption studies.

Process	Parameters			Plot	Additional Information	
**Adsorption**	effects of HCl, HNO_3_ concentration	*m* = 0.5 ± 0.0005 g, *C*_0_ = 100 mg M(II)/L, *V* = 0.05 L, *V_as_* = 180 rpm, *A* = 8, *T* = 293 K, *t* = 1 min–4 h		*q_t_* vs. *t*	Pd(II) S1–S9	0.1–6.0 mol/L HCl—100 mg M(II)/L; 0.1–0.9 mol/L HCl—0.9–0.1 mol/L HNO_3_—100 mg M(II)/L
		*S_b_* = 0.300–1.2 mm Diaion™ CR20	*S_b_* = 0.315–1.25 mm Lewatit^®^ VP OC 1065		Cu(II) S1 *–S9 *	
**Kinetics**	initial Pd(II) concentration	*m* = 0.5 ± 0.0005 g, *C*_0_ = 100, 500 mg/L, 1000 mg/L, *V* = 0.05 L, *V_as_* = 180 rpm, *A* = 8, *T* = 293 K, *t* = 1 min–4 h		*q_t_* vs. *t*	0.1 mol/L HCl—x mg Pd(II)/L; 0.9 mol/L HCl–0.1 mol/L HNO_3_—x mg Pd(II)/L, x—100, 500 mg/L, 1000 mg/L	
		*S_b_* = 0.300–1.2 mm Diaion™ CR20	*S_b_* = 0.315–1.25 mm Lewatit^®^ VP OC 1065			
	agitation speed	*m* = 0.5 ± 0.0005 g, *C*_0_ = 500 mg/L, *V* = 0.05 L, *V_as_* = 120, 150, 180 rpm, *A* = 8, *T* = 293 K, *t* = 1 min–4 h		*q_t_* vs. *t*	0.1 mol/L HCl—500 mg Pd(II)/L; 0.9 mol/L HCl–0.1 mol/L HNO_3_—500 mg Pd(II)/L	
		*S_b_* = 0.300–1.2 mm Diaion™ CR20	*S_b_* = 0.315–1.25 mm Lewatit^®^ VP OC 1065			
	bead size	*m* = 0.5 ± 0.0005 g, *C*_0_ = 500 mg/L, *V* = 0.05 L, *V_as_* = 180 rpm, *A* = 8, *T* = 293 K		*q_t_* vs. *t*	0.1 mol/L HCl—500 mg Pd(II)/L; 0.9 mol/L HCl–0.1 mol/L HNO_3_—500 mg Pd(II)/L	
		*t* = 1 min–4 h for f2, f3, *t* = 4 h for f1, f4, f5 Diaion™ CR20	*t* = 1 min–4 h for f1, f2, f3, *t* = 4 h for f4, f5 Lewatit^®^ VP OC 1065			
	temperature	*m* = 0.5 ± 0.0005 g, *C*_0_ = 500 mg/L, *V* = 0.05 L, *V_as_* = 180 rpm, *A* = 8, *T* = 293, 313, 333 K		*q_t_* vs. *t*	0.1 mol/L HCl—500 mg Pd(II)/L; 0.9 mol/L HCl–0.1 mol/L HNO_3_—500 mg Pd(II)/L	
		*S_b_* = 0.300–1.2 mm Diaion™ CR20	*S_b_* = 0.315–1.25 mm Lewatit^®^ VP OC 1065			
**Isotherm**	initial M(II) concentration	*m* = 0.5 ± 0.0005 g, *C*_0_ = 1–5000 mg/L, *V* = 0.05 L, *V_as_* = 180 rpm, *A* = 8, *T* = 293 K, *t* = 24 h		*q_e_* vs. *C_e_*	0.1 mol/L HCl—x mg M(II)/L; Pd(II) *C*_0_ = 100–5000 mg/L; Ni(II), Zn(II) *C*_0_ = 20–500 mg/L; Cu(II) *C*_0_ = 1–80 mg/L.	
		*S_b_* = 0.300–1.2 mm Diaion™ CR20	*S_b_* = 0.315–1.25 mm Lewatit^®^ VP OC 1065			
**Desorption**	eluting agent type and concentration	*m* = 0.5 ± 0.0005 g, *V* = 0.05 L, *V_as_* = 180 rpm, *A* = 8, *T* = 293 K, *t* = 4 h, eluting agents: 0.1, 1, 2 mol/L HNO_3_, HCl, NH_3_∙H_2_O, NaOH, H_2_SO_4_		%*S* or %*D* vs. *C_o_* and type	Adsorption S: 0.1 mol/L HCl—100 mg Pd(II)/L; 6 mol/L HCl—100 mg Cu(II)/L; Desorption D: 0.1, 1, 2 mol/L HNO_3_, HCl, NH_3_∙H_2_O, NaOH, H_2_SO_4_	
		*S_b_* = 0.300–1.2 mm Diaion™ CR20	*S_b_* = 0.315–1.25 mm Lewatit^®^ VP OC 1065			

*m*—the mass of ion exchanger, *C*_0_—the initial M(II) concentration, *V*—the volume of the solution, *V_as_*—the agitation speed, *A*—the amplitude, *T*—the temperature, *t*—the time, *S_b_*—the whole bead size distribution, * 1.2 mm > *f*1 ≥ 0.75 mm, 0.75 > *f*2 ≥ 0.6 mm, 0.6 mm > *f3* ≥ 0.43 mm, 0.43 > *f4* ≥ 0.385 mm, *f5* < 0.385 mm.

## Data Availability

Data are included in the paper and in Supplementary Materials.

## Supplementary Materials

The following supporting information can be downloaded at: https://www.mdpi.com/article/10.3390/molecules29184386/s1, Figure S1: Comparison of M(II) adsorption efficiency for Diaion™ CR20 (a,c,e) and Lewatit^®^ VP OC 1065 (b,d,f) for the HCl-HNO_3_ systems; Figure S2. Comparison of M(II) complexes in the chloride solutions; Figure S3. Schematic representation of sieve analysis of ion exchangers (a), comparison of mass of fractions (b) and the fractions obtained for Diaion™ CR20 (c); Figure S4. Comparison of the breakthrough curves of Cu(II) ions adsorption on Lewatit^®^ VP OC 1065 (a,c) and Diaion™ CR20 (b,d) from the chloride 0.1–6 mol/L HCl—100 mg Cu(II)/L (a,b) and the chloride-nitrate(V) solutions 0.1–0.9 mol/L HCl—0.9–0.1 mol/L HNO_3_—100 mg Cu(II)/L (c,d); Figure S5: Comparison of the adsorption and desorption of Cu(II) ions on/from Diaion™ CR20. Table S1: Effect of temperature on Cu(II) adsorption on different adsorbents; Table S2: Kinetic parameters for the Pd(II) adsorption on the ion exchangers from the 0.1–6 mol/L HCl—100 mg/L systems using the PFO and IPD models; Table S3: Isotherm parameters for the Cu(II) ions adsorption on the Diaion™ CR20 and Lewatit^®^ VP OC 1065 ion exchangers.

## References

[1] Elshkaki A., Graedel T.E., Ciacci L., Reck B.K. (2016). Copper demand, supply, and associated energy use to 2050. Glob. Environ. Change.

[2] Galos K., Smakowski T., Galos K., Smakowski T. (2014). Wstępna propozycja metodyki identyfikacji surowców kluczowych dla polskiej gospodarki. Zesz. Nauk. Inst. Gospod. Surowcami Miner. Energi Pol. Akad. Nauk..

[3] Bobba S., Carrara S., Huisman J., Mathieux F., Pavel C. (2020). Critical Raw Materials for Strategic Technologies and Sectors in the EU A Foresight Study. Tech. Rep..

[4] Chen Y., Qiao Q., Cao J., Li H., Bian Z. (2021). Precious metal recovery. Joule.

[5] Watari T., Nansai K., Nakajima K. (2021). Major metals demand, supply, and environmental impacts to 2100: A critical review. Resour. Conserv. Recycl..

[6] Ahirwar R., Tripathi A.K. (2021). E-waste management: A review of recycling process, environmental and occupational health hazards, and potential solutions. Environ. Nanotechnol. Monit. Manag..

[7] McCarthy S., Braddock D.C., Wilton-Ely J.D.E.T. (2021). Strategies for sustainable palladium catalysis. Coord. Chem. Rev..

[8] Liu G., Wu Y., Tang A., Pan D., Li B. (2020). Recovery of scattered and precious metals from copper anode slime by hydrometallurgy: A review. Hydrometallurgy.

[9] Briffa J., Sinagra E., Blundell R. (2020). Heavy metal pollution in the environment and their toxicological effects on humans. Heliyon.

[10] Lee J., Kurniawan, Hong H.-J., Chung K.W., Kim S. (2020). Separation of platinum, palladium and rhodium from aqueous solutions using ion exchange resin: A review. Sep. Purif. Technol..

[11] Ajiboye E.A., Aishvarya V., Petersen J. (2023). Selective recovery of copper from the mixed metals leach liquor of e-waste materials by ion-exchange: Batch and column study. Minerals.

[12] Xu B., Chen Y., Zhou Y., Zhang B., Liu G., Li Q., Yang Y., Jiang T. (2022). A Review of Recovery of Palladium from the Spent Automobile Catalysts. Metals.

[13] Krstić V., Urošević T., Pešovski B. (2018). A review on adsorbents for treatment of water and wastewaters containing copper ions. Chem. Eng. Sci..

[14] Gupta A., Sharma V., Sharma K., Kumar V., Choudhary S., Mankotia P., Kumar B., Mishra H., Moulick A., Ekielski A. (2021). A review of adsorbents for heavy metal decontamination: Growing approach to wastewater treatment. Materials.

[15] Fei Y., Hu Y.H. (2022). Design, synthesis, and performance of adsorbents for heavy metal removal from wastewater: A review. J. Mater. Chem. A.

[16] Tofan L., Wenkert R. (2020). Chelating polymers with valuable sorption potential for development of precious metal recycling technologies. Rev. Chem. Eng..

[17] Kołodyńska D. (2009). Chelating ion exchange resins in removal of heavy metal ions from waters and wastewaters in presence of a complexing agent. Przem. Chem..

[18] Sud D., Dr I., Luqman M. (2012). Chelating Ion Exchangers: Theory and Applications. Ion Exchange Technology I.

[19] Sengupta A.K., Zhu Y., Hauze D. (1991). Metal(II) ion binding onto chelating exchangers with nitrogen donor atoms: Some new observations and related implications. Environ. Sci. Technol..

[20] Afolabi F.O., Musonge P., Bakare B.F. (2022). Adsorption of Copper and Lead Ions in a Binary System onto Orange Peels: Optimization, Equilibrium, and Kinetic Study. Sustainability.

[21] Zaimee M.Z.A., Sarjadi M.S., Rahman M.L. (2021). Heavy Metals Removal from Water by Efficient Adsorbents. Water.

[22] Product Data Sheet Lewatit VP OC 1065. https://lanxess.com/en-us/products-and-brands/products/l/lewatit--vp-oc-1065.

[23] Product Data Sheet DiaionTM CR20. https://www.diaion.com.tw/upload-files/pdf/CR20.pdf.

[24] Wójcik G. (2019). Sorption and reduction of chromium ions by the chelating ion exchanger Diaion CR20. Physicochem. Probl. Miner. Process..

[25] Alesi W.R., Kitchin J.R. (2012). Evaluation of a Primary Amine-Functionalized Ion-Exchange Resin for CO_2_ Capture. Ind. Eng. Chem Res..

[26] Wołowicz A., Wawrzkiewicz M., Hubicki Z. (2018). Toxic Heavy Metal Ions and Metal-Complex Dyes Removal from Aqueous Solutions Using an Ion Exchanger and Titanium Dioxide. Fibres Text. East. Eur..

[27] Fiol N., Villaescusa I. (2008). Determination of sorbent point zero charge: Usefulness in sorption studies. Environ. Chem. Lett..

[28] Kosmulski M. (2023). The pH dependent surface charging and points of zero charge. X. Update. Adv. Colloid Interface Sci..

[29] Giacomni F., Menegazzo M.A.B., da Silva M.G., da Silva A.B., Dornellas de Barros M.A.S. (2017). Importância da determinação do ponto de carga zero como característica de tingimento de fibras proteicas [Point of zero charge of protein fibers, an important characteristic for dyeing]. Matéria.

[30] Kosmulski M. (2009). Surface Charging and Points of Zero Charge.

[31] Sarbak Z. (2000). Adsorption and Adsorbents: Theory and Application.

[32] Ling P., Liu F., Li L., Jing X., Yin B., Chen K., Li A. (2010). Adsorption of divalent heavy metal ions onto IDA-chelating resins: Simulation of physicochemical structures and elucidation of interaction mechanisms. Talanta.

[33] Bąk J., Sofińska-Chmiel W., Gajewska M., Malinowska P., Kołodyńska D. (2023). Determination of the Ni(II) Ions Sorption Mechanism on Dowex PSR2 and Dowex PSR3 Ion Exchangers Based on Spectroscopic Studies. Materials.

[34] Shi J., Yi S., He H., Long C., Li A. (2013). Preparation of Nanoscale Zero-Valent Iron Supported on Chelating Resin with Nitrogen Donor Atoms for Simultaneous Reduction of Pb^2+^ and NO_3_^−^. Chem. Eng. J..

[35] Kołodyńska D., Fila D., Hubicki Z. (2020). Recovery of Lanthanum(III) and Nickel(II) Ions from Acidic Solutions by the Highly Effective Ion Exchanger. Molecules.

[36] Zong L., Liu F., Chen D., Zhang X., Ling C., Li A. (2018). A Novel Pyridine Based Polymer for Highly Efficient Separation of Nickel from High-Acidity and High-Concentration Cobalt Solutions. Chem. Eng. J..

[37] Zagorodni A.A., Kotova D.L., Selemenev V.F. (2002). Infrared Spectroscopy of Ion Exchange Resins: Chemical Deterioration of the Resins. React. Funct. Polym..

[38] Kononova O.N., Duba E.V., Shnaider N.I., Pozdnyakov I.A. (2017). Ion exchange extraction of platinum(IV) and palladium(II) from hydrochloric acid solutions. Russ. J. Appl. Chem..

[39] Bernardis F.L., Grant R.A., Sherrington D.C. (2005). A review of methods of separation of the platinum-group metals through their chloro-complexes. React. Funct. Polym..

[40] Nikoloski A.N., Ang K.-L. (2013). Review of the Application of Ion Exchange Resins for the Recovery of Platinum-Group Metals from Hydrochloric Acid Solutions. Miner. Process. Extr. Metall. Rev..

[41] Gala A., Sanak-Rydlewska S. (2010). Sorpcja jonów metali toksycznych z roztworów wodnych na odpadach naturalnych—Przegląd literaturowy. Górnictwo Geoinżynieria.

[42] Ampiaw R.E., Lee W. (2020). Persimmon tannins as biosorbents for precious and heavy metal adsorption in wastewater: A review. Int. J. Environ. Sci. Technol..

[43] Wołowicz A., Hubicki Z. (2010). Selective adsorption of palladium(II) complexes onto the chelating ion exchange resin Dowex M 4195—Kinetic studies. Solv. Extr. Ion Exch..

[44] Wołowicz A., Hubicki Z. (2012). The use of the chelating resin of a new generation Lewatit MonoPlus TP-220 with the bis-picolylamine functional groups in the removal of selected metal ions from acidic solutions. Chem. Eng. J..

[45] Yang B., Tong X., Deng Z., Lv X. (2016). The Adsorption of Cu Species onto Pyrite Surface and Its Effect on Pyrite Flotation. J. Chem..

[46] Islam M.A., Angove M.J., Morton D.W. (2019). Macroscopic and modeling evidence for nickel(II) adsorption onto selected manganese oxides and boehmite. J. Water Proc. Eng..

[47] El-Taib Heakal F., Abd-Ellatif W.R., Tantawy N.S., Taha A.A. (2018). Impact of pH and temperature on the electrochemical and semiconducting properties of zinc in alkaline buffer media. RSC Adv..

[48] Nguyen V.N.H., Song S.J., Lee M.S. (2022). Selective Precipitation of Ammonium Hexachloropalladate from leaching solutions of cemented palladium with zinc. J. Min. Metall. Sect. B Metall..

[49] Önder T., Wang X., Zimmermann P., Burheim O.S., Deng L. (2024). Tailoring anion exchange membranes for palladium recovery from industrial solutions using electrodialysis. Chem. Eng. J..

[50] Kononova O.N., Duba E.V., Medovikov D.V., Krylov A.S. (2018). Ion-Exchange Sorption of Palladium(II) from Hydrochloric Acid Solutions in the Presence of Silver(I). Russ. J. Phys. Chem..

[51] Kononova O.N., Mikhaylova N.V., Melnikov A.M., Kononov Y.S. (2011). Ion exchange recovery of zinc from chloride and chloride–sulfate solutions. Desalination.

[52] Wołowicz A., Wawrzkiewicz M. (2021). Screening of Ion Exchange Resins for Hazardous Ni(II) Removal from Aqueous Solutions: Kinetic and Equilibrium Batch Adsorption Method. Processes.

[53] Chouchane T., Boukari A., Khireddine O., Chibani S., Chouchane S. (2023). Equilibrium, kinetics, and thermodynamics of batch adsorption of Mn(II) ions on blast furnace slag (BFS) and kaolin (KGA). J. Eng. Appl. Sci..

[54] Kakavandi B., Kalantary R.R., Farzadkia M., Mahvi A.H., Esrafili A., Azari A., Yari A.R., Javid A.B. (2014). Enhanced chromium(VI) removal using activated carbon modified by zero valent iron and silver bimetallic nanoparticles. J. Environ. Health Sci. Eng..

[55] Wołowicz A., Hubicki Z. (2012). Ion Exchange Recovery of Palladium(II) from Acidic Solutions Using Monodisperse Lewatit SR-7. Ind. Eng. Chem. Res..

[56] Wołowicz A., Hubicki Z. (2016). Sorption behavior of Dowex PSR-2 and Dowex PSR-3 resins of different structures for metal(II) removal. Solvent Extr. Ion Exch..

[57] Wołowicz A., Hubicki Z. (2014). Adsorption characteristics of noble metals on the strongly basic anion exchanger Purolite A-400TL. J. Mater. Sci..

[58] Wołowicz A., Hubicki Z. (2012). Applicability of new acrylic, weakly basic anion exchanger Purolite A-830 of very high capacity in removal of palladium(II) chloro-complexes. Ind. Eng. Chem. Res..

[59] Omidinasab M., Rahbar N., Ahmadi M., Kakavandi B., Ghanbari F., Kyzas G.Z., Martinez S.S., Jaafarzadeh N. (2018). Removal of vanadium and palladium ions by adsorption onto magnetic chitosan nanoparticles. Environ. Sci. Pollut. Res..

[60] Wołowicz A., Hubicki Z. (2016). Carbon-based adsorber resin Lewatit AF 5 applicability in metal ion recovery. Micropor. Mesopor. Mater..

[61] Tran H.N., You S.-J., Hosseini-Bandegharaei A., Chao H.-P. (2017). Mistakes and inconsistencies regarding adsorption of contaminants from aqueous solutions: A critical review. Water Res..

[62] Alghamdi A.A., Al-Odayni A.B., Saeed W.S., Al-Kahtani A., Alharthi F.A., Aouak T. (2019). Efficient adsorption of lead(II) from aqueous phase solutions using polypyrrole-based activated carbon. Materials.

[63] Scheffler A. (1996). Lewatit-MonoPlus. The Latest Generation of Monodisperse Ion Exchange Resin with Outstanding Properties for Optimizing Water Treatment System.

[64] Dubois I., Holgersson S., Allars S., Malmström M.E., Birkle P., Torres-Alvarado I.S. (2010). Correlation between Particle Size and Surface Area for Chlorite and K-feldspar. Water-Rock Interaction.

[65] Liu Y., Miao J., Han H., Xu P. (2021). Differences in Influence of Particle Size on the Adsorption Capacity between Deformed and Undeformed Coal. ACS Omega.

[66] Ten Hulscher T.E.M., Cornelissen G. (1996). Effect of temperature on sorption equilibrium and sorption kinetics of organic micropollutants—A review. Chemosphere.

[67] Sánchez J.M., Hidalgo M., Salvadó V. (2000). The separation of Au(III) and Pd(II) in hydrochloric acid solutions by strong anion type II exchange resin: The effect of counter ion concentration and temperature. Solvent Extr. Ion Exch..

[68] Patel H. (2020). Batch and continuous fixed bed adsorption of heavy metals removal using activated charcoal from neem (*Azadirachta indica*) leaf powder. Sci. Rep..

[69] Wasewar K.L. (2010). Adsorption of metals onto tea factory waste: A review. Int. J. Res. Rev. Appl. Sci..

[70] Jachuła J., Kołodyńska D., Hubicki Z. (2011). Sorption of Cu(II) and Ni(II) ions in the presence of the methylglycinediacetic acid by microporous ion exchangers and sorbents from aqueous solutions. Open Chem..

[71] Shen C., Chang Y., Fang L., Min M., Xiong C.H. (2016). Selective removal of copper with polystyrene–1,3-diaminourea chelating resin: Synthesis and adsorption studies. New J. Chem..

[72] Bień T., Kołodyńska D., Franus W. (2021). Functionalization of Zeolite NaP1 for Simultaneous Acid Red 18 and Cu(II) Removal. Materials.

[73] Ciesielczyk F., Bartczak P., Klapiszewski Ł., Jesionowski T. (2017). Treatment of model and galvanic waste solutions of copper(II) ions using a lignin/inorganic oxide hybrid as an effective sorbent. J. Hazard. Mater..

[74] Lagergren S. (1898). About the theory of so-called adsorption of soluble substances. K. Sven. Vetenskapsakademiens Handl..

[75] Ho Y.S., McKay G. (1999). Pseudo-second order model for sorption processes. Process Biochem..

[76] Weber W.J., Morris J.C. (1963). Kinetics of adsorption on carbon from solution. J. Sanit. Eng. Div..

[77] Tan K.L., Hameed B.H. (2017). Insight into the adsorption kinetics models for the removal of contaminants from aqueous solutions. J. Taiwan Inst. Chem. Eng..

[78] Marković D.D., Lekić B.M., Rajaković-Ognjanović V.N., Onjia A.E., Rajaković L.V. (2014). A new approach in regression analysis for modeling adsorption isotherms. Sci. World. J..

[79] Sivarajasekar N., Baskar R. (2019). Adsorption of Basic Magenta II onto H_2_SO_4_ activated immature Gossypium hirsutum seeds: Kinetics. isotherms. mass transfer. thermodynamics and process design. Arab. J. Chem..

[80] Qiu H., Lv L., Pan B.C., Zhang Q.J., Zhang W.M., Zhang Q.X. (2009). Critical review in adsorption kinetic models. J. Zhejiang Univ. Sci. A.

[81] Aydin S., Kajjumba G.W., Emik S., Öngen A., Kurtulus Özcan H., Aydın S., Edebali S. (2018). Modelling of adsorption kinetic processes-Errors, theory and application. Advanced Sorption Process Applications.

[82] Langmuir I. (1918). The adsorption of gases on plane surfaces of glass, mica and platinum. J. Am. Chem. Soc..

[83] Freundlich H.M.F. (1906). Over the adsorption in solution. J. Phys. Chem..

[84] Wang J., Guo X. (2020). Adsorption isotherm models: Classification, physical meaning, application and solving method. Chemosphere.

[85] Chen X., Hossain M.F., Duan C., Lu J., Tsang Y.F., Islam M.S., Zhou Y. (2022). Isotherm models for adsorption of heavy metals from water—A review. Chemosphere.

[86] Parodi A., Vincent T., Pilsniak M., Trochimczuk A.W., Guibal E. (2008). Palladium and platinum binding on an imidazol containing resin. Hydrometallurgy.

[87] Nikoloski A.N., Ang K.-L., Li D. (2015). Recovery of platinum, palladium and rhodium from acidic chloride leach solution using ion exchange resins. Hydrometallurgy.

[88] Agarwal B., Balomajumder C., Thakur P.K. (2013). Simultaneous co-adsorptive removal of phenol and cyanide from binary solution using granular activated carbon. Chem. Eng. J..

[89] Istratie R., Stoia M., Păcurariu C., Locovei C. (2019). Single and simultaneous adsorption of methyl orange and phenol onto magnetic iron oxide/carbon nanocomposites. Arab. J. Chem..

[90] Rakitskaya T.L., Vasylechko V.O., Kiose T.A., Dzhyga G.M., Gryshchouk G.V., Volkova V.Y. (2017). Some features of Pd(II) and Cu(II) adsorption on bentonites. Ads. Sci. Technol..

[91] Huang Z., Wang C., Zhao J., Wang S., Zhou Y., Zhang L. (2020). Adsorption behavior of Pd(II) ions from aqueous solution onto pyromellitic acid modified-UiO-66-NH_2_. Arab. J. Chem..

