# Supplementary material for: Evaluation of Adsorption Ability of Lewatit® VP OC 1065 and Diaion™ CR20 Ion Exchangers for Heavy Metals with Particular Consideration of Palladium(II) and Copper(II)
Source: Molecules. 2024 Sep 15;29(18):4386. doi: 10.3390/molecules29184386 (PMC11434107; doi:10.3390/molecules29184386)
Supplement: Supplementary file 1 [file molecules-29-04386-s001.zip › molecules-3149255-supplementary.pdf]

molecules

Evaluation of adsorption ability of Lewatit® VP OC 1065 and Diaion™ CR20 ion exchangers for heavy metals with particular consideration palladium(II) and copper(II)

Anna Wołowicz*, Zbigniew Hubicki

Department of Inorganic Chemistry, Faculty of Chemistry, Institute of Chemical Sciences, Maria Curie Sklodowska University, Maria Curie-Sklodowska Square 2, 20-031 Lublin, Poland

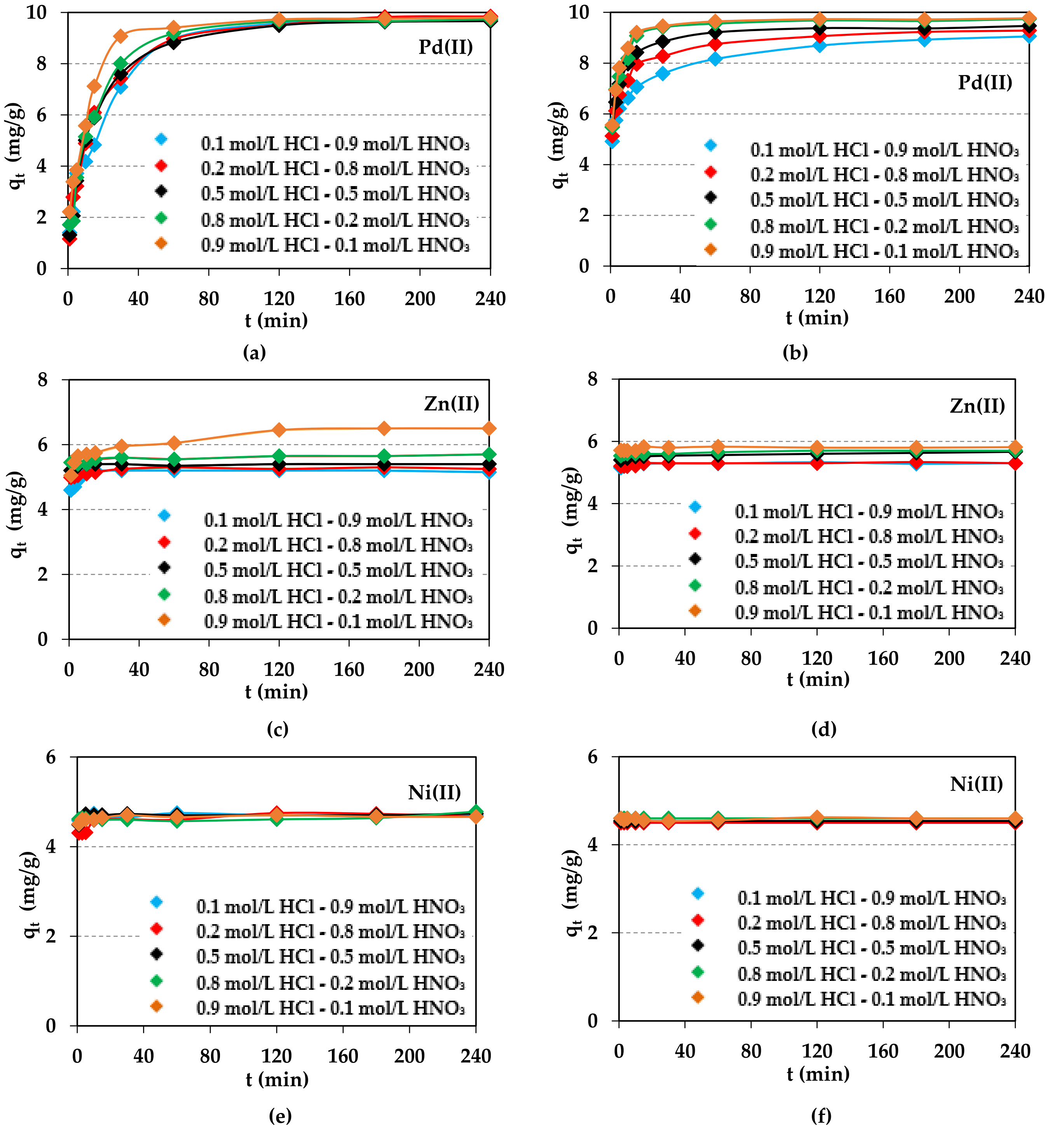

Figure S1. Comparison of M(II) adsorption efficiency for Diaion™ CR20 **(a,c,e)** and Lewatit® VP OC 1065 **(b,d,f)** for the HCl-HNO_3 systems.

* Corresponding author: Tel.: +48 81 537 57 27
E-mail address: anna.wolowicz@mail.umcs.pl

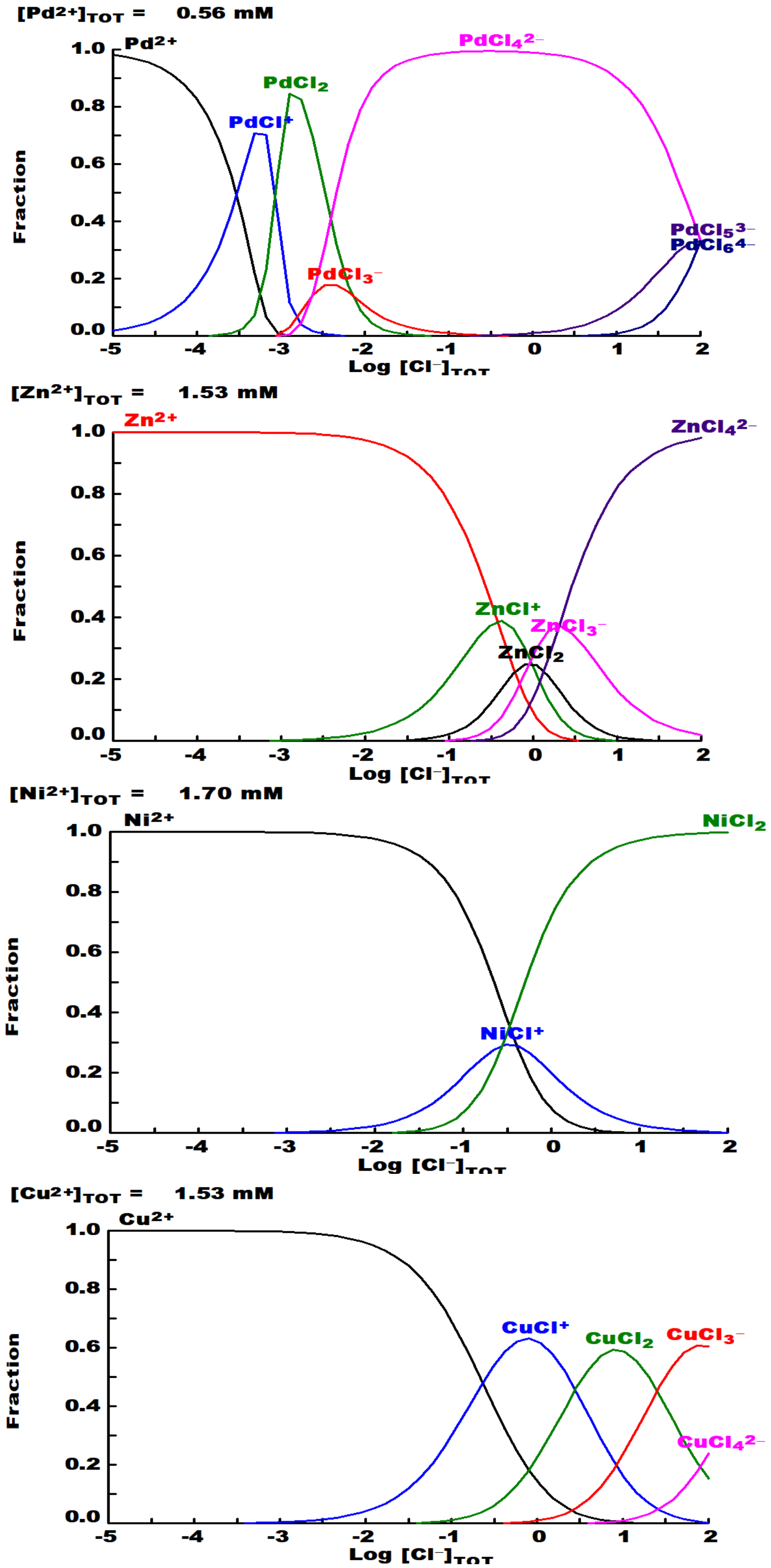

Figure S2. Comparison of M(II) complexes in the chloride solutions.

(a)

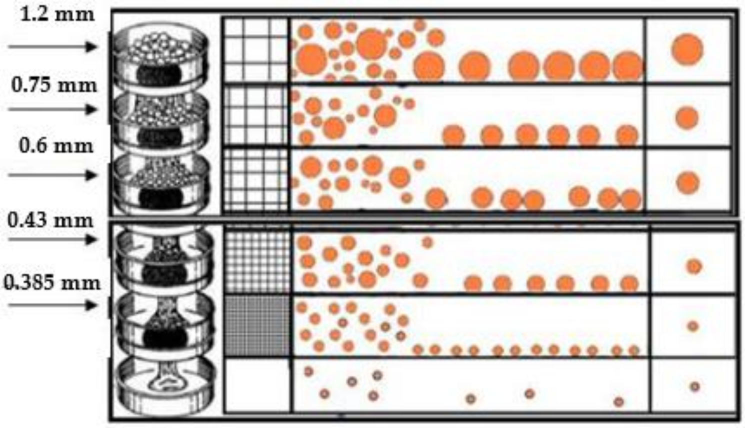

(b)

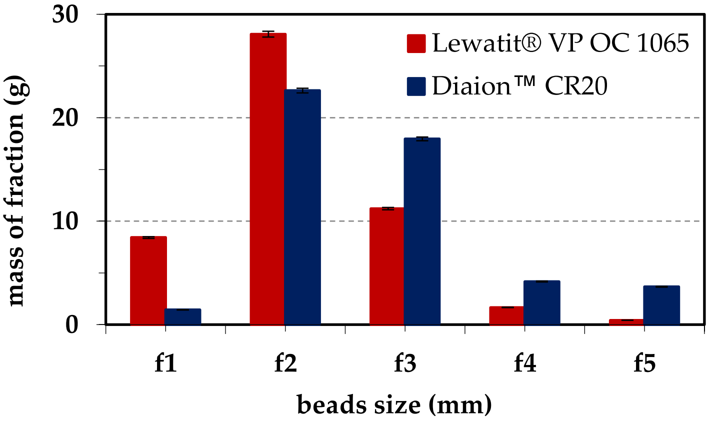

(c)

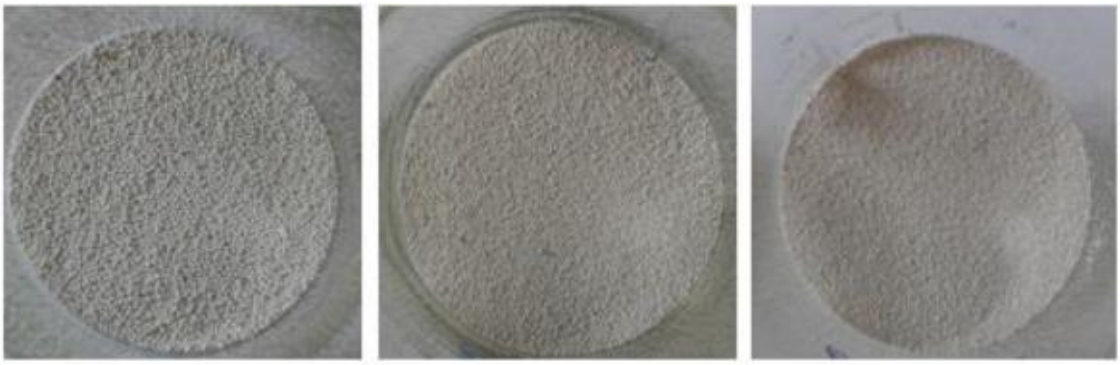

0.75 mm ≤ f1 < 1.2 mm   0.6 mm ≤ f2 < 0.75 mm   0.43 mm ≤ f3 < 0.6 mm

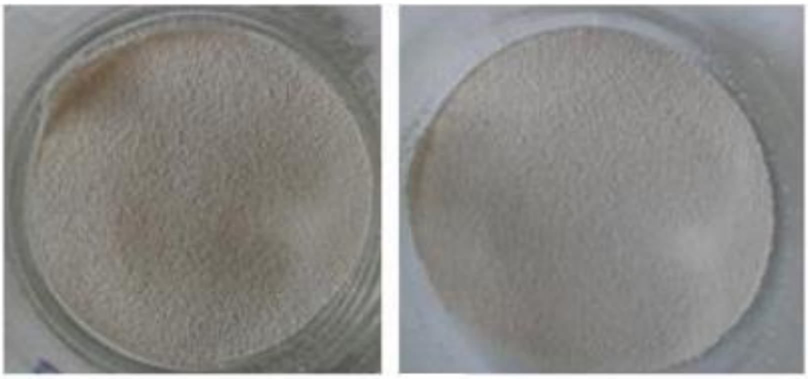

0.385 mm ≤ f4 < 0.43 mm   f5 < 0.385 mm

Figure S3. Schematic representation of sieve analysis of ion exchangers **(a)**, comparison of mass of the fractions **(b)** and fractions obtained for Diaion™ CR20 **(c)**.

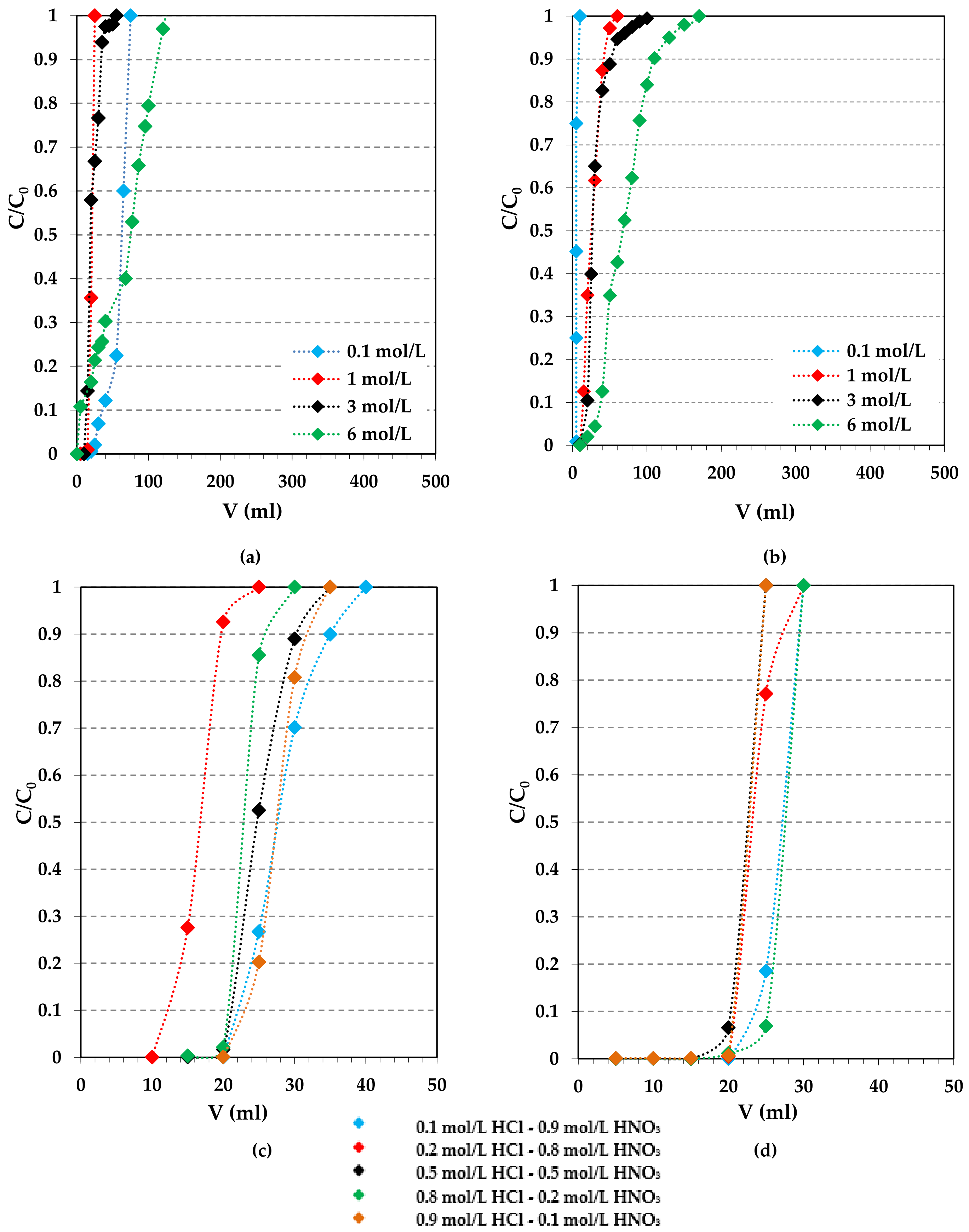

Figure S4. Comparison of the breakthrough curves of Cu(II) ions adsorption on Lewatit® VP OC 1065 **(a,c)** and Diaion™ CR20 **(b,d)** from the chloride 0.1–6 mol/L HCl–100 mg Cu(II)/L **(a,b)** and the chloride-nitrate(V) solutions 0.1–0.9 mol/L HCl—0.9–0.1 mol/L HNO_3–100 mg Cu(II)/L **(c,d).**

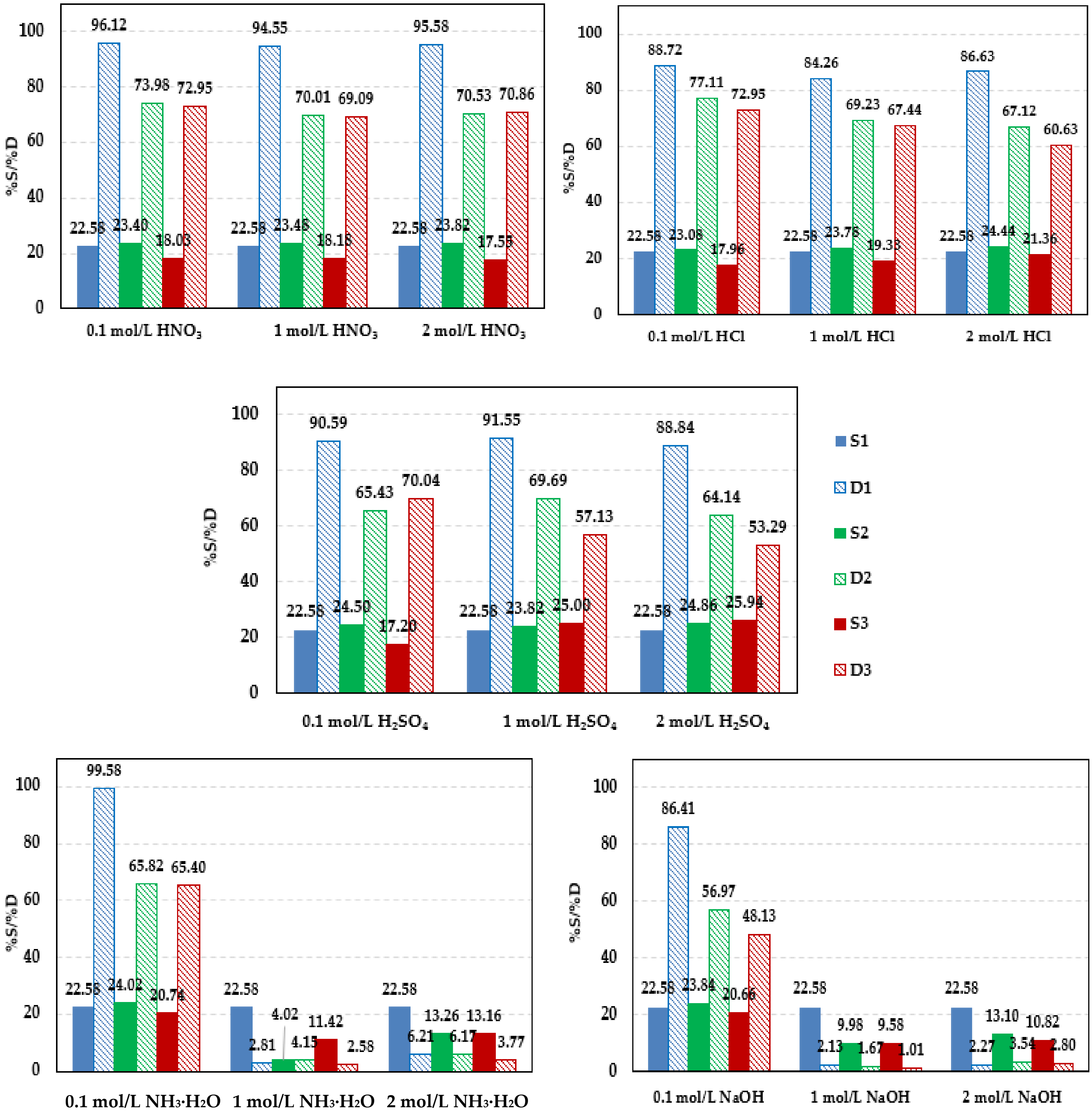

Figure S5. Comparison of the adsorption and desorption of Cu(II) ions on/from Diaion™ CR20.

Table S1. Effect of temperature on Cu(II) adsorption on different adsorbents.

Adsorbent; adsorbat	Temperature (K)	Effect, adsorption capacity (q_e, mg/g) and/or percentage removal (%R)	Ref.
Lewatit MonoPlus M 500, SBA<br>Lewatit MonoPlus M 600, SBA<br>Lewatit MP 62, WBA;<br>Cu(II)-methylglycinediacetic acid (MGDA) complexes	293, 303, 313, 323, 333	A slight increase in the Cu(II)-MGDA complexes adsorption with the increasing T<br>$\Delta H_0>0$, $\Delta S_0>0$, $\Delta G_0>0$<br>endothermic, nonspontaneous<br>ΔS_0 indicate that there is an increase in the degree of randomness during the sorption process	[70]
polystyrene–1,3-diaminourea chelating resin<br>Cu(II)	288, 298, 308	the equilibrium adsorption increased over the range of reaction T from about 150 to 180 mg/g<br>$\Delta H_0>0$, $\Delta S_0>0$, $\Delta G_0<0$<br>endothermic, spontaneous	[71]
Zeolite NaP1<br>Cu(II)	293, 313, 333	better adsorption at higher temperature<br>$\Delta H_0>0$, $\Delta S_0>0$, $\Delta G_0<0$<br>endothermic, spontaneous	[72]
lignin/MgO-SiO_2 hybrid sorbent<br>Cu(II)	298, 303, 308, 313	T does not have a significant effect<br>the highest %R=99.9% at 25 °C<br>$\Delta H_0<0$, $\Delta S_0<0$, $\Delta G_0<0$, T↑ ΔG_0↑<br>exothermic, spontaneous, ΔS_0 point to complex interactions between adsorbent and adsorbate	[73]

T— the temperature, ΔH_0 (kJ/mol)—the enthalpy, ΔG_0 (kJ/mol)—the free energy, ΔS_0 (J/mol·K)—the entropy, SBA— the strongly basic anion exchanger, WBA—the weakly basic anion exchanger

Table S2. Kinetic parameters for the Pd(II) adsorption on the ion exchangers from the 0.1—6 mol/L HCl—100 mg/L systems using the PFO and IPD models.

**Parameters**		**Diaion™ CR20**				**Lewatit® VP OC 1065**			
		**0.1 mol/L HCl**	**1 mol/L HCl**	**3 mol/L HCl**	**6 mol/L HCl**	**0.1 mol/L HCl**	**1 mol/L HCl**	**3 mol/L HCl**	**6 mol/L HCl**
$q_{e\,exp}$ (mg/g)		9.90	9.90	7.70	5.90	9.97	9.38	7.29	5.89
**PFO LN**	$q_{e\,cal}$ (mg/g)	3.08	0.98	1.68	0.39	0.12	0.65	1.04	0.41
	k_1 (1/min)	0.294	0.020	0.027	0.004	0.028	0.031	0.027	0.006
	R^2	0.992	0.652	0.934	0.723	0.491	0.748	0.931	0.803
**PFO Non—LR**	$q_{e\,cal}$ (mg/g)	9.71	9.18	6.57	6.57	9.76	9.03	6.28	6.28
	k_1 (1/min)	0.499	0.339	0.246	0.160	1.055	0.426	0.472	0.160
	R^2	0.971	0.918	0.840	0.842	0.876	0.962	0.653	0.560
	R^2adj	0.962	0.894	0.794	0.797	0.840	0.952	0.554	0.434
	$MPSD$	0.018	0.097	0.212	2.569	0.019	0.032	0.272	9.414
**IPD**	$q_{e\,cal}$ (mg/g)	10.38	12.55	10.90	7.60	10.16	10.74	9.68	9.38
	k_i (mg/g min$^{0.5}$)	0.06	0.34	0.46	0.32	0.02	0.17	0.33	0.52
	R^2	0.640	0.781	0.974	0.767	0.608	0.942	0.965	0.954
	R^2adj	0.537	0.719	0.966	0.701	0.496	0.926	0.955	0.941

Table S3. Isotherm parameters for the Cu(II) ions adsorption on the Diaion™ CR20 and Lewatit® VP OC 1065 ion exchangers.

Model	Parameters	Diaion™ CR20	Lewatit® VP OC 1065
		Cu(II)	Cu(II)
$q_{e\ exp}$ (mg/g)		0.11	1.04
**Linear regression**			
Langmuir	Q_0 (mg/g)	0.23	1.24
	k_L (L/mg)	0.011	1.062
	R^2	0.269	0.972
Freundlich	k_F (mg$^{1-1/n}$ L$^{1/n}$/g)	0.004	0.530
	$1/n$	0.798	0.548
	R^2	0.825	0.969
**Non-linear regression**			
Langmuir	Q_0 (mg/g)	0.02	1.02
	k_L (L/mg)	1.176	1.449
	$MPSD$	4.720	0.196
	R^2	0.732	0.952
	R^2_{adj}	0.655	0.927
Freundlich	k_F (mg$^{1-1/n}$ L$^{1/n}$/g)	0.002	0.514
	$1/n$	0.916	0.541
	$MPSD$	1.110	0.116
	R^2	0.823	0.946
	R^2_{adj}	0.772	0.919
